# Supplementary material for: Probabilistic analysis of spatial viscoelastic cues in 3D cell culture using magnetic microrheometry
Source: Biophys J. 2024 Dec 16;124(2):351–62. doi: 10.1016/j.bpj.2024.12.010 (PMC11788488; doi:10.1016/j.bpj.2024.12.010)
Supplement: Document S1. Figures S1–S16 and Tables S1–S3 [file mmc1.pdf]

Biophysical Journal, Volume *124*

Supplemental information

Probabilistic analysis of spatial viscoelastic cues in 3D cell culture using magnetic microrheometry

Ossi Arasalo, Arttu J. Lehtonen, Mari Kielosto, Markus Heinonen, and Juho Pokki

S1 Supplemental methods

S1.1 Cell viability

We quantified the viability of the initial measurement condition after 1 day of incubation, using Cyto3D Live–Dead Assay. The cells' viability is $96.6 \pm 2.2\%$ (mean ± standard deviation, 5 measurements with altogether 3603 cells, and 721 cells/measurement on average). We have subsequently controlled that, in the following conditions (after 2 and 3 days), the cells remain spread with almost no exceptions (indicating continued fibroblast viability).

S1.2 Magnetic microrheometry-related biophysics

The magnetic-probe displacement is related to linear viscoelasticity as in the following equation described in [1].

$$6\pi r\eta\dot{x}(t) + 6\pi r\mu x(t) = M\nabla BV = V\underbrace{M\nabla B_0}_{f_v}\sin(\omega t) \tag{S1}$$

$$x(t) = \frac{2f_v r^2}{9|G^*|}sin(2\pi ft - \phi) \tag{S2}$$

where x is the probe displacement, $\dot{x}$ is the probe velocity, r is the probe radius, η is dynamic viscosity, μ is the shear modulus, M is the probe magnetization, V is the probe volume and ∇B is the magnetic field gradient strength. We have applied time-dependent sinusoidal forces with an amplitude of ∇B_0. For convenience, the probe magnetization and the unknown amplitude are fused into a single calibration constant, the volumetric force, f_v, which is estimated during the system calibration. Solving the differential equation in Eq. S1 results in Eq. S2. Instead of estimating the values of η and μ, we have estimated the complex shear modulus, $G^* = G' + iG''$, where the storage modulus is $G' = \mu$, and the loss modulus is $G'' = \omega\eta$. In this work, we have reported the absolute complex shear modulus $|G^*|$ as a measure of stiffness, and the phase angle ϕ of the complex shear modulus, as a measure of viscous energy dissipation (i.e. the liquid-like characteristics).

S1.3 Bayesian modeling method for estimation of spatially varying viscoelasticity

Here, the hierarchical prior for the noise term (σ_i), which is independent for each magnetic probe, is defined:

$$\sigma_\mu \sim \mathcal{N}(0, 1) \tag{S3}$$

$$\sigma_\sigma, l_\sigma \sim \text{Half-}\mathcal{N}(0, 1) \tag{S4}$$

$$\sigma_i \sim \text{Inverse Gamma}(\sigma_\mu, \sigma_\sigma) \tag{S5}$$

Then, the hierarchical prior for the mean behavior, where we use non-centered parametrization for the viscoelasticity terms, is defined for the absolute complex shear modulus and the phase angle:

$$\mu_{|G*|} \sim \mathcal{N}\left(\mathcal{N}(50, 15), \text{Half-}\mathcal{N}(0, 1)\right) \tag{S6}$$

$$\mu_\phi \sim \mathcal{N}\left(\mathcal{N}(0, 0.1), \text{Half-}\mathcal{N}(0, 1)\right) \tag{S7}$$

Table S1: Number of measurements in different conditions for 3D CAF-cell cultures

Condition	# Repetition	Incubation [# Day]	Probes	Fields of view
CAF	1	1	39	7
Contr	1	1	34	4
CAF	1	2	56	7
Contr	1	2	34	4
CAF	1	3	44	6
Contr	1	3	20	3
CAF	2	1	45	6
Contr	2	1	51	6
CAF	2	2	46	6
Contr	2	2	58	6
CAF	2	3	60	6
Contr	2	3	55	6
CAF	3	1	51	6
Contr	3	1	54	6
CAF	3	2	38	6
Contr	3	2	63	6
CAF	3	3	39	6
Contr	3	3	60	6

S1.4 System calibration

The full calibration model is used as follows:

$$x_i(t) \sim \mathcal{N}\left(-\frac{2}{9\eta\omega} r^{*2} f_v \left(\cos(\omega t - \phi_i) - 1\right) + \alpha_i t + \beta_i, \sigma\right) \tag{S8}$$
$$\sigma \sim \text{Inverse Gamma}(\alpha = 5, \beta = 5) \tag{S9}$$
$$r_\mu \sim \mathcal{N}(6, 1) \tag{S10}$$
$$r_\sigma \sim \text{Inverse Gamma}(\alpha = 2, \beta = 0.5) \tag{S11}$$
$$r^* \sim \mathcal{N}(r_\mu, r_\sigma) \tag{S12}$$
$$r \sim \mathcal{N}(r^*, \tau) \tag{S13}$$
$$f_v \sim \mathcal{N}(280000, 14000) \tag{S14}$$
$$\alpha, \phi \sim \mathcal{N}(0, 0.1) \tag{S15}$$
$$\beta \sim \mathcal{N}(0, 3.) \tag{S16}$$
$$\eta = 1.000074433378914 \tag{S17}$$
$$\omega = 0.05(2\pi) \tag{S18}$$

The calibration model has the same formula as described in the main article text with the addition of the parameter ϕ to handle small miscalibrations, and the letters α_i and β_i to capture small linear drifts in the data. The integration constant C is found using the initial guess of $x(t = 0) = 0$.

The prior for f_v, the primary output of the calibration model, shows minimal sensitivity to prior choices. As long as the mean and scale of the prior cover the true value, all choices converge to the same result, well within the standard deviation reported in the main text.

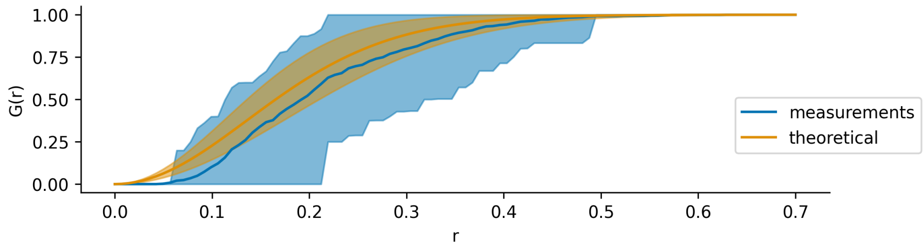

Figure S1: Ripley's G-function calculated for the magnetic probe locations. Comparison is done against homogeneous Poisson point process $G(r) = 1 - e^{-\lambda\pi r^2}$ (where $\lambda = n/A$, n is number of cells in a FOV and A is the area of a FOV and r is the normalized distance). Intervals clearly overlap meaning no strong indications of clustering or aggregation.

Further details on prior sensitivity of τ are included in the subsection S2.1. These results also apply to the current calibration model.

S2 Supplemental results

S2.1 Sensitivity analysis

We performed a sensitivity analysis to test how different priors affect the model's results. Our focus was on the priors most important for stability of the viscoelastic field estimates and the radius correction. By varying these priors, we evaluated their impact on the stability of the results.

Correctly inferring posteriors for the magnitude of heterogeneity (α_μ) and heterogeneity differences between FOVs (α_σ) in $|G^*|$ fields is important from the perspective of interpretability. Too restrictive prior choices (wrongly informative) can lead to incorrect interpretation of the parameters while still providing stable estimates for the viscoelastic fields shown in the left column of Fig. S2. The top row in the figure (Fig. S2A) shows the FOV-specific magnitude of the heterogeneity with the global mean and standard deviation removed ($\alpha_{z[i]}$ in non-centered parametrization $\alpha_{[i]} = \text{Softplus}(\alpha_\mu + \alpha_\sigma \alpha_{z[i]})$). This $\alpha_{z[i]}$ shows how too low α_μ values result in a positive bias. Now, too low global magnitude, in Fig. S2B, is partially explained by the bias and increased FOV variability (α_σ). When the scale parameter of the priors are increased the true region of the heterogeneity is covered properly and differences in the prior choices become unsensitive. We also verified if changes in the hyperprior of the $\mu_{|G^*|}$ scale affects the inference. In theory, this parameter can lead to oversmoothing in the Gaussian process, because the spatial variability could be explained by highly varying mean level having a large uncertainty. However, as evident from the rightmost column in Fig. S2, that change is redundant as it does not affect α_μ or α_σ noticeably.

Hence, as long as the chosen prior has thick enough 'tails' to capture the true scale of the heterogeneity, in our case in the order of 10 Pascals, model becomes unsensitive to the prior choices. Student's $\text{t}(3, 0, 20)$ for the α_μ with $\mathcal{N}(0, 5)$ for α_σ have scale parameters which are applicable to capture the heterogeneity of the measured collagen matrices.

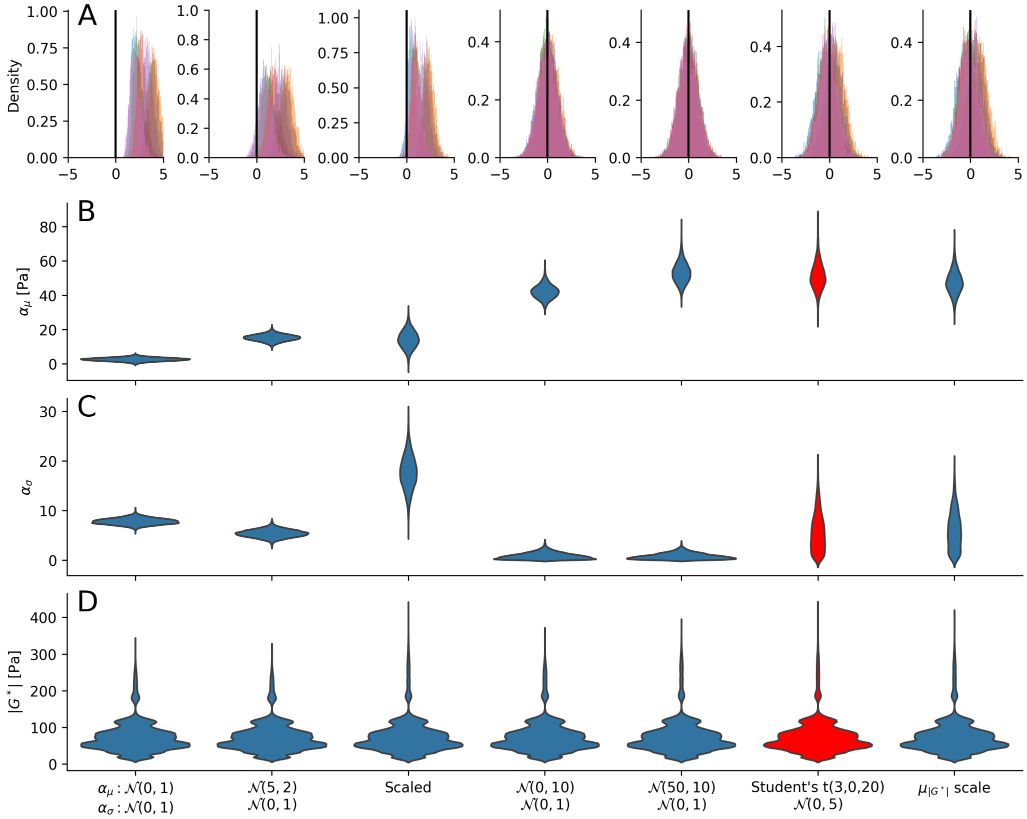

Figure S2: Evaluation of the tested priors is visualized, and the chosen priors are indicated with red color. **(A)** Posterior distributions of $\alpha_{\mathrm{z[j]}}$ with different priors for α_μ and α_σ. A positive bias is induced if the prior is too restrictive that is visible clearly for $\mathcal{N}(0,1)$. Instead, the priors with longer 'tails' for α_μ (e.g. $\mathcal{N}(0,10)$ or $\mathcal{N}(50,10)$) lead to the desired $\alpha_{z[j]}$ remaining at zero and having no bias. In 'Scaled', a multiplication by 10 is applied to the original priors ($\alpha_\mu \sim \mathcal{N}(0,1)$ and $\alpha_\sigma \sim \mathcal{N}(0,1)$). In '$\mu_{|G^*|}$ scale', hierarchical hyperprior for the scale of mean level, $\mathcal{N}(0,1)$ is increased to $N(0,5)$. **(B)** Identifiably of α_μ (magnitude of heterogeneity) with the different priors. When the scale increases the value is identified correctly. **(C)** Effects of the priors to the heterogeneity between microscopy fields of view (FOVs). **(D)** Posterior of $|G^*|$ across over all locations and spatial points. While the interpretation changes, estimated viscoelastic field stays similar suggesting the model is robust to different choices of priors.

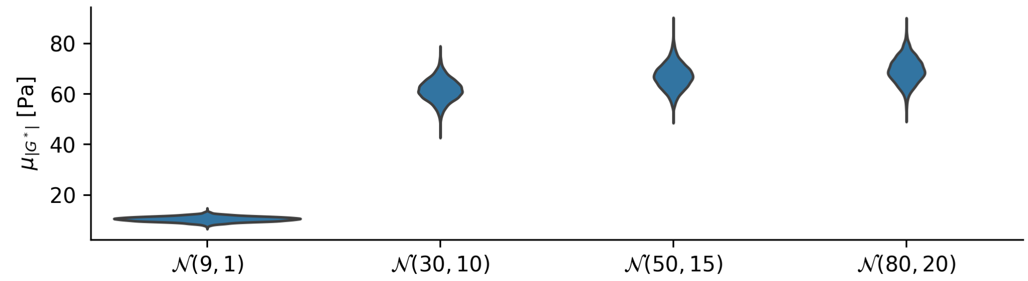

Figure S3: Effects of the prior choice in $\mu_{|G^*|}$. The model is robust unless the prior is highly misspecified as in $\mathcal{N}(9, 1)$.

Kernel length scale for the $|G^*|$ field does not suffer from similar challenges in the interpretability as we are more interested in good fit rather than understanding the dynamics in the light of length scale. In short, length scale is more limited by the distance of the magnetic probes. If we have multiple FOVs with different 'closeness' of the probes, the interpretability is difficult as the FOVs with more spread out probe distribution has less information about the fast variations in the material. Therefore, comparison becomes difficult. The choice of the hierarchical prior is also not important as long as it captures the size of the measurement window. The coordinates are normalized between $[0, 1]$, so the choice of Half-$\mathcal{N}(0, 1)$ captures possible variation for both $|G^*|$ and ϕ.

We also tested how the model behaves if we change the prior for the $|G^*|$ offset $\mu_{|G|^*}$. The results are visible in Fig. S3, which shows that the choice of $\mathcal{N}(50, 15)$ is reasonable as the actual data mean is within the prior. If this is highly misspesified, such as $\mathcal{N}(9, 1)$, estimated mean level is simply wrong and the interpretation of the value would be different. In that case to be avoided, the mean of the data is partially explained by the amplitude of the Gaussian processes which is unwanted behavior for interpretability. Obviously, if this model would be used for stiffer materials, the mean prior should be scaled accordingly to contain the true mean or alternatively the whole amplitude could be scaled based on the data so more 'default' priors could be used.

The phase angle ϕ, the second viscoelasticity parameter, is insensitive to the choices of priors for the kernel amplitude parameter. The main reason for this is that the ϕ's domain is finite ($[0, \pi/2]$), which is not the case for the domain of $|G^*|$ (i.e. $\mathbb{R}^+$). Thus, the possible heterogeneity of ϕ is limited to

a smaller range which can be covered with simple choices such as $\mathcal{N}(0, 0.5)$. We found that if the prior is far away from zero (positive or negative), the Sigmoid function squashes the values and too much mass is put to either having a zero heterogeneity, or toward the absolute maximum. MCMC can slow down considerably and computational diagnostics can report divergent transitions indicating poor exploration of the parameter space. A possible reason for such behaviors is the promotion of unrealistically large values which are close to the saturating parts of the Sigmoid function. This behavior is visible if the scale is ≥ 1.

On the other hand, for the mean level μ_ϕ, we iteratively test different choices of priors shown in Fig. S4. Posterior distributions of ϕ fields remain unaffected the but interpretability of specific parameters can be changed if the prior is poorly chosen. Narrow priors such as $\mathcal{N}(0, 0.1)$ are too restrictive and might not capture the true mean value and result in increased scale of the mean level (Fig. S4B). Additionally, the transformation $f(x) = \arcsin(\text{Sigmoid}(x))$ used to map the real axis to $[0, \pi/2]$ transform the mean of 0 to ≈ 28 degrees which means the prior is biasing towards unintended value. Increasing the scale captures the true value as visible in Fig. S4A.

As pointed out in the main text, we want to promote elastic like properties because collagen cultures are mostly elastic. This can be achieved by giving negative mean values, because they are transformed to approximately 0. As shown in S4, these choices are irrelevant in identifying the parameters. The prior recommended in the main text is chosen because of the ease in interpretation. Here the transformation of the parameter is characterized by the prior choice of $\mathcal{N}(0, 0.3)$, meaning we are defining the prior in the transformed space. This choice is purely for the benefit of understandability, it does not have any practical implications as shown in Fig. S4. Similarly, if we would like to promote viscous properties the prior could be changed to $\mathcal{N}(\pi/2, 0.3)$ which is difficult to define in the original space.

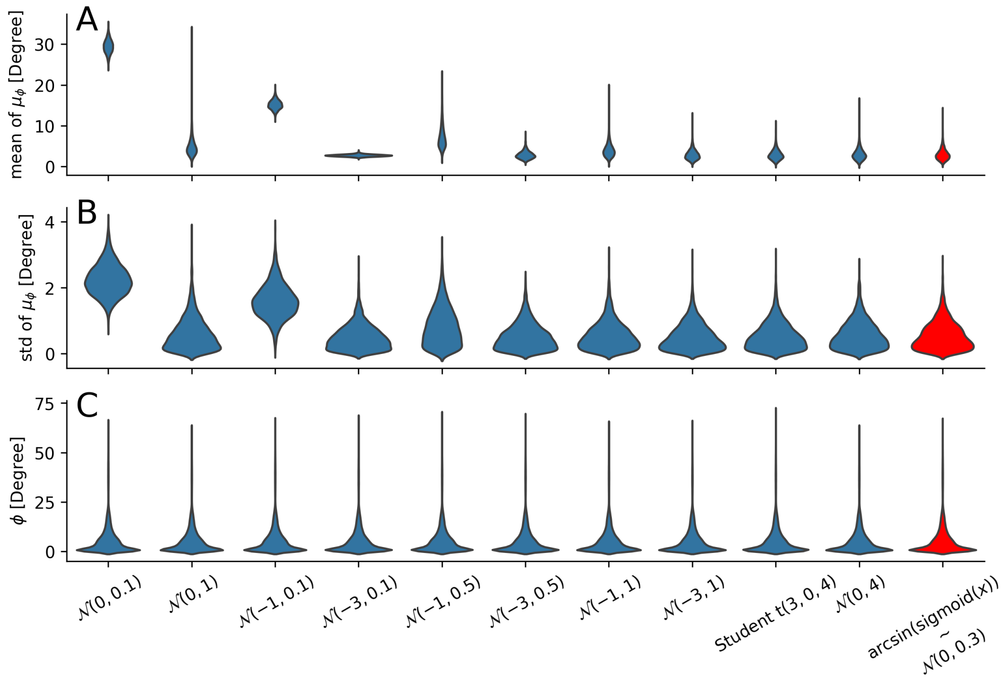

Figure S4: Sensitivity of the estimated phase angle's (ϕ) mean level to priors of the mean level. The estimated standard deviation of the mean level and ϕ fields are also shown. (**A**) Posterior of the mean-level parameter μ_ϕ. As long as the width of the distribution is sufficient, the correct level is inferred insensitively. (**B**) shows how misspecifications lead to positive bias in the mean standard deviation parameter. (**C**) Posterior distributions remain unaffected as a function of the priors. The red color highlights the updated choice of the prior ($\arcsin(\text{sigmoid}(\mu_\phi)) \sim \mathcal{N}(0, 0.3)$), based on the sensitivity analysis. All priors except for the updated prior are defined in the untransformed space. Some of the priors have therefore negative mean values as they are transformed to the domain of ϕ after sampling.

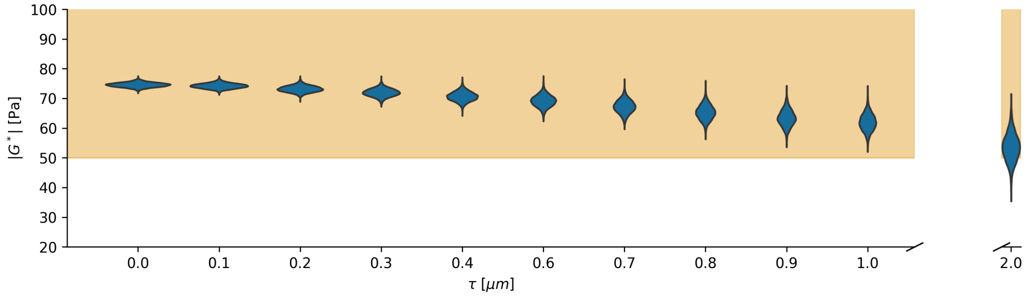

Figure S5: Sensitivity of modeled $|G^*|$ to varied τ. Blue violinplots indicate posterior distributions of the mean. The orange shading shows the region between the data's 25th to 75th percentiles.

Next, we focus on the sensitivity analysis of r^* to τ as it controls how large errors are allowed which in turn can overly smooth the results due to squared relation to the amplitude of the signal. We fit the model at values between $[0, 2]$ which equal at maximum of $\approx \pm 6$ pixels of error to a one representative measurement condition to evaluate variability. As evident from the Fig. S5A, increasing τ increases the uncertainty (width of the distributions) while decreasing the mean. At large values of τ the model has more flexibility to solve the inverse problem and due to the squared relationship of the radius estimate, spatial variability in the viscoelasticity is now explained by highly varied magnetic bead size. Finally, as τ increases to unrealistically large values, the model approaches $|G^*|$ prior $\mu_{|G|*} \sim \mathcal{N}(50, 15)$.

The choice of 0.1 used in this paper is reasonable as it is insensitive to changing the radius values, while still capturing possible uncertainty in the radius estimate. However, as shown, this is an influential parameter so care must be taken. For example, domain expertize of the used imaging system shouldn't be downplayed either. If the imaging quality is poor (large τ), or alternatively, probe size would be much smaller (larger contribution from τ), this parameter would dominate the results and the final spatial fields would be smoother. In a way this is also useful behavior as it reflects the uncertainty of the measurement system more accurately, and should raise concerns of applicability of the measurement device and the collected data. In our case the choice of 0.1 reflects our prior understanding of the radius estimate accuracy in our imaging system and is shown to have only a small effect to the final results.

S2.2 Posterior checks

Figure S9 shows how the model differs from the traditional data analysis pipeline. In Fig. S9A,B we see how the model and the data (traditional analysis pipeline) follow each other closely. However, the uniqueness of different FOVs (violinplots' irregular shapes) is more pronounced as the model is less subject to noise in measurements and captures viscoelasticity more robustly.

Figure S9C indicates how much the model is recalibrating the uncertain radius values. As specified by the prior choice of 0.1 for τ, changes are small. Further, the shape is symmetric suggesting that the radius estimates are not biased which gives some further evidence that the algorithm for calculating the radius values is working similarly across samples.

The level of difference between the model and the traditional analysis pipeline are shown in Figs. S9D and E. Differences are mostly in the range of few Pascals and $\pm$ 2 degrees. These findings further support the findings in Figs. S9A and B how to model matches closely what we are expecting to get from the data.

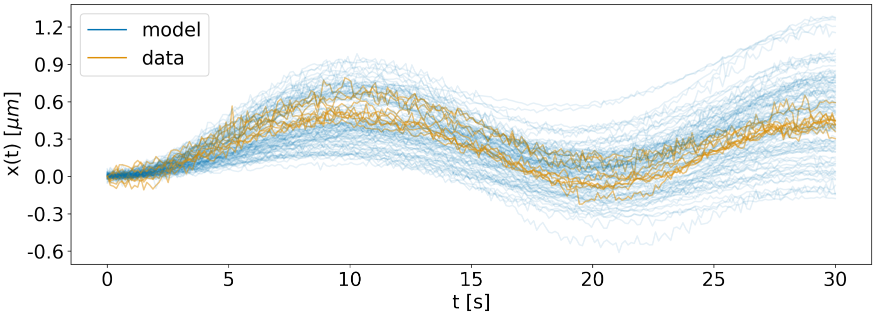

Figure S6: Draws from the calibration model's prior distribution, compared to the raw probe displacement data.

A more detailed visualization of the differences between the model and the traditional analysis pipeline are shown in Fig. S10. It shows how the inferred viscoelastic properties are similar to the averages of different magnetic-reference probe pairs, which is the intended behavior. Further, large outliers are mostly rejected resulting in longer tails (higher uncertainty). If the number of reference probes is only one, we see more averaging behavior with wider distributions. As the model has less information for that particular probe it relies more on the pooled information from the hierarchical priors and shape of the field generated by the Gaussian process.

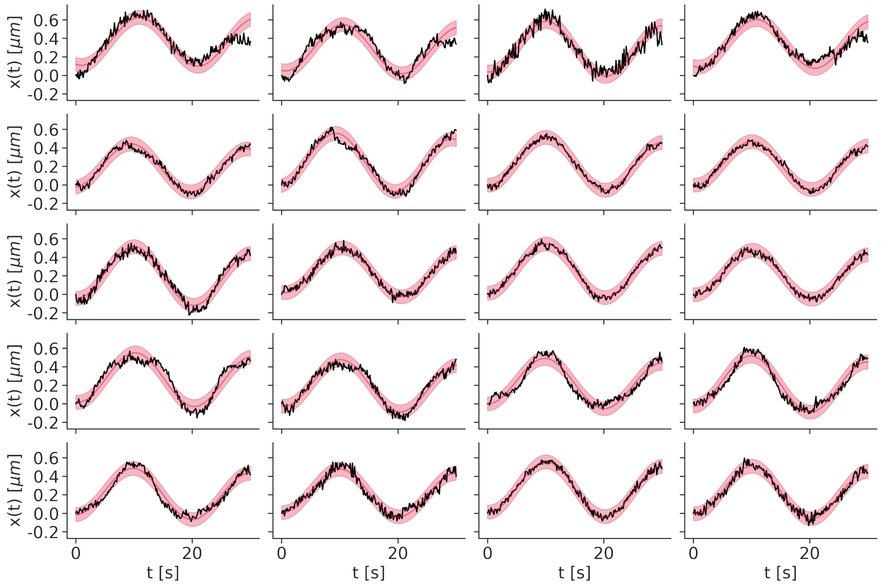

Figure S7: Posterior predictive checks of the measured probe displacement signals in the calibration data (showing half of the signals). Shaded regions represent the 95% credible intervals.

S2.3 Additional results

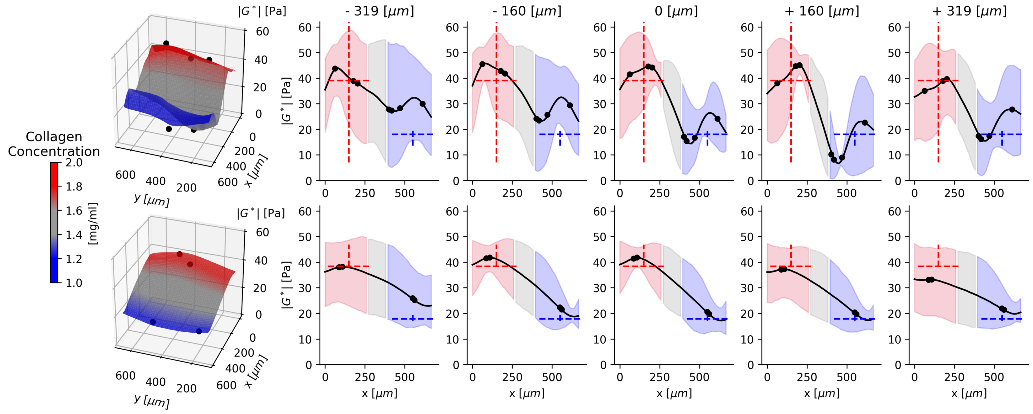

Figure S8: Supplementary visualization of stiffness gradient at varied off-axis positions, relative to the centerpoint of the y axis, in Fig. 3 of the manuscript.

Table S2: Probabilistic differences in viscoelasticity's mean levels ($\mu_{|G^*|}$, and μ_ϕ) in comparisons between each 3D cultures condition and its controls. Shaded rows show the differences over all repetitions in a condition. Values as bolded indicate significance using a threshold value of 0.95.

Incubation day	Repetition ID	Stiffness [%] $P(\mu_{|G^*|}^{\text{CAF}} > \mu_{|G^*|}^{\text{control}})$	Phase angle [%] $P(\mu_\phi^{\text{CAF}} < \mu_\phi^{\text{control}})$
1	1	**99.75**	58.42
1	2	**99.98**	**96.4**
1	3	**98.15**	**97.4**
1		**99.29**	84.08
2	1	**99.08**	73.07
2	2	**99.78**	47.97
2	3	**99.95**	**99.92**
2		**99.60**	73.66
3	1	13.53	82.82
3	2	85.83	54.02
3	3	**99.93**	93.35
3		66.43	76.73

Table S3: Probabilistic differences in viscoelasticity's mean levels ($\mu_{|G^*|}$, and μ_ϕ) in comparisons between different days of incubation for the 3D cultures.

Incubation days	Stiffness [%] $P(\mu_{|G^*|}^{\text{CAF day n}} < \mu_{|G^*|}^{\text{CAF day n+1}})$	Phase angle [%] $P(\mu_\phi^{\text{CAF day n}} < \mu_\phi^{\text{CAF day n+1}})$
1 & 2	71.94	66.08
1 & 3	74.73	67.86
2 & 3	52.76	47.32

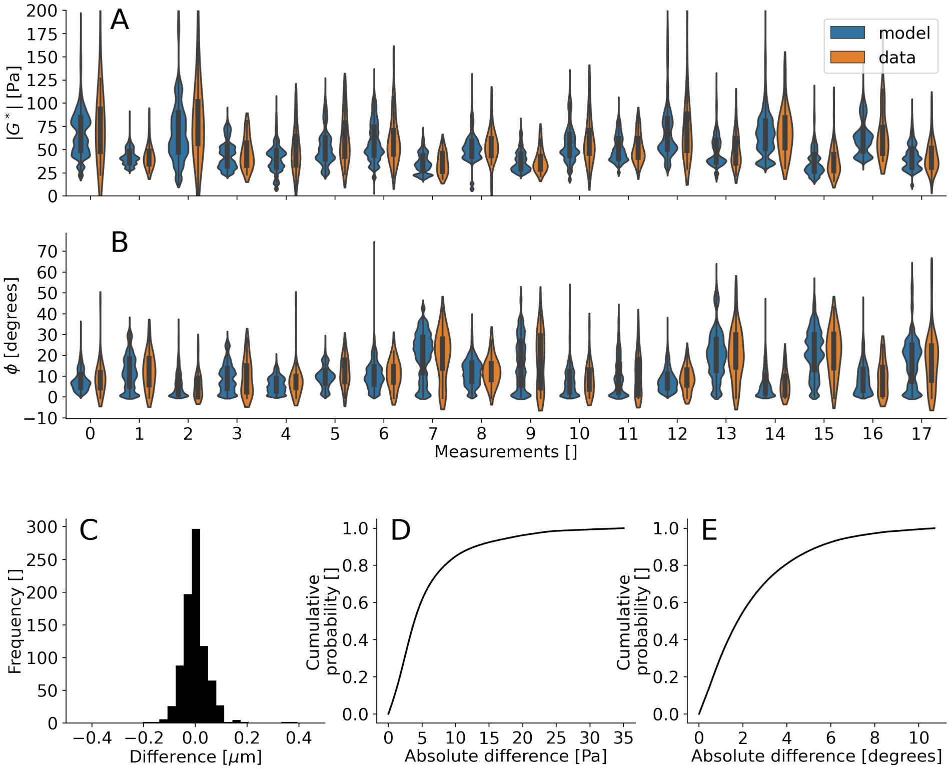

Figure S9: Posterior validation. **(A)** and **(B)** show a comparison of estimated viscoelastic properties calculated from the raw measurements and the model. The different sets of measurements, noted as 'Measurements' in the X axes (with all magnetic probes over multiple FOVs within a single violinplot) are shown. **(C)** shows the magnitude of the radius adjustments done by the measurement error model. **(D)** and **(E)** show the cumulative empirical probability distributions of the absolute differences in the viscoelastic estimates (raw data and the model) for the absolute complex shear modulus and the phase angle, respectively.

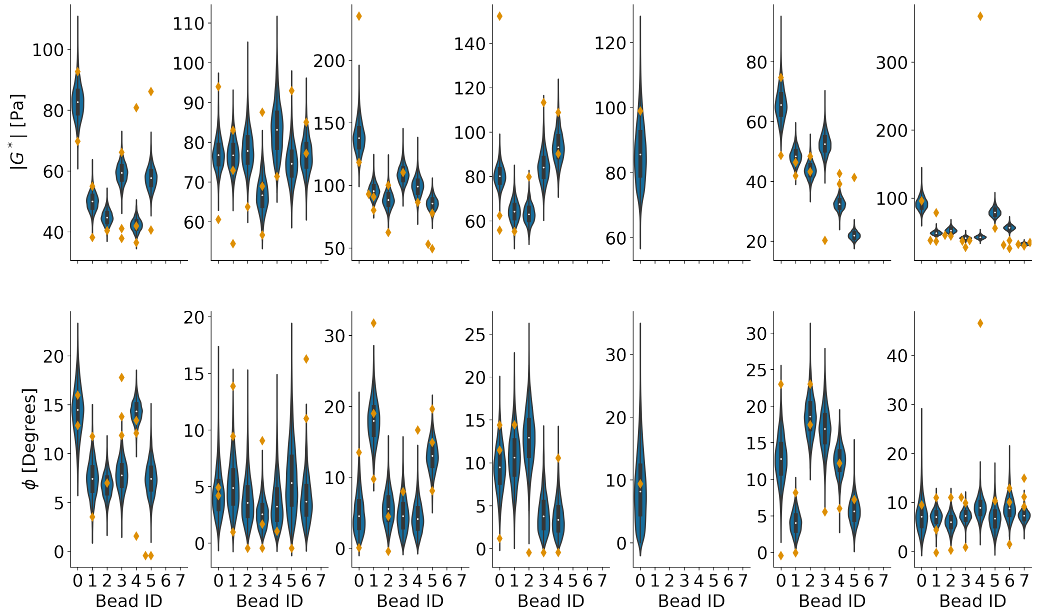

Figure S10: Comparison of estimated viscoelastic properties calculated from the raw measurements and the model outputs from a single sample holder for measurements. Orange triangles represent estimates calculated using conventional pipeline, and blue violin plots show the same value estimated with the Gaussian processes. Different columns are different measured FOVs and the X axes distinguish the different used magnetic probes.

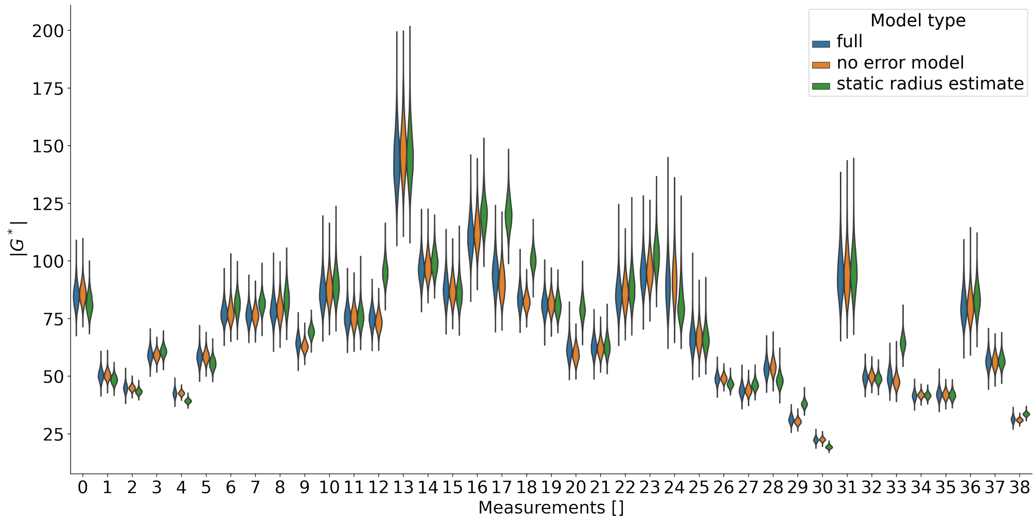

Figure S11: Model comparisons in respect to inclusion of the radius estimation in the model. The X axis shows different measured probes and the Y axis indicates the absolute complex shear modulus. Different colors show different versions of the spatial model. The 'full' model is the proposed model, the 'no error model' uses the raw measurements instead of the measurement error model, and the 'static radius estimate' uses the mean probe radius of 6.14 μm. The changes in the model are highly overlapping indicating that the measurement error model does not shift values unrealistically.

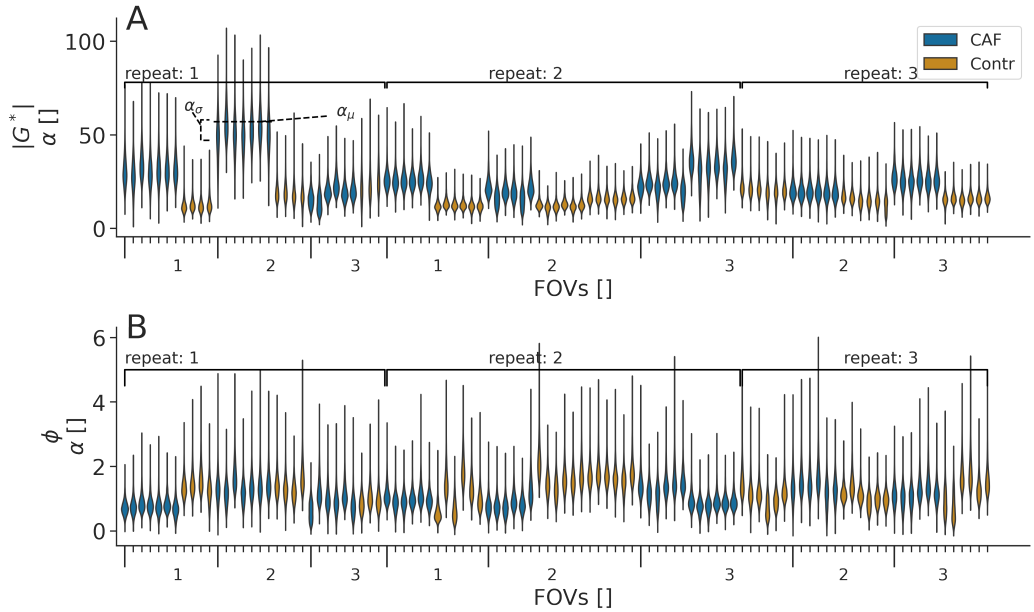

Figure S12: Estimate of heterogeneity in viscoelasticity in respect to varied microscopy FOV. Y axes show the magnitude of the heterogeneity in the **(A)** absolute complex shear modulus and the **(B)** phase angle. Minor ticks in the X axis represent the individual FOVs while the major ticks are corresponding the incubation day number. The meanings of α_μ and α_σ for single measurement conditions are depicted visually.

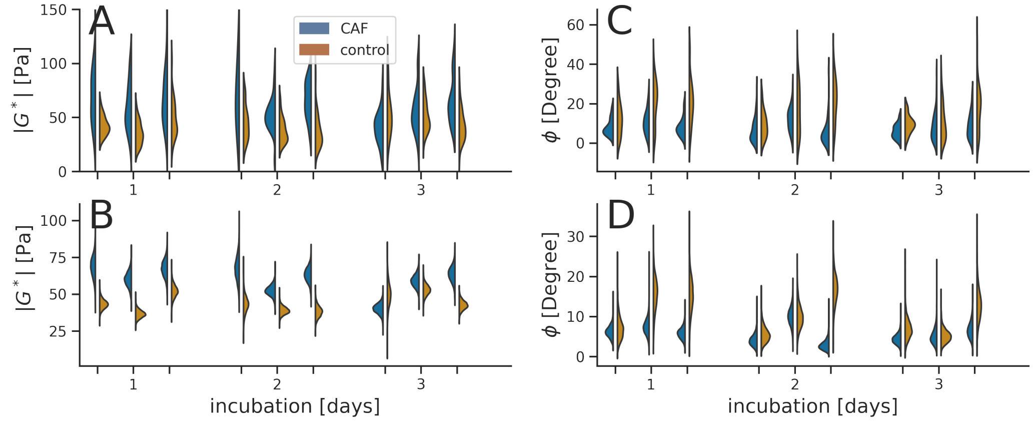

Figure S13: Posterior distributions for each measurement condition plotted. A and C are the full posteriors while B and D are the mean offsets. Minor ticks indicate different repeations.

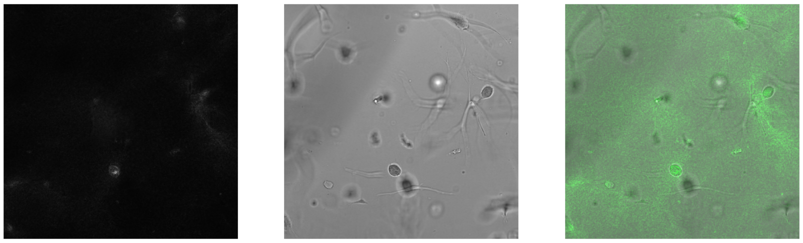

Figure S14: Unprocessed images of collagen fibers (reflectance, left) and breast-cancer-associated fibroblasts (brightfield, middle), and these images having overlaid (right). The overlaid image has been postprocessed using contrast limited adaptive histogram equalization (CLAHE). The images' width/height is 581 μm.

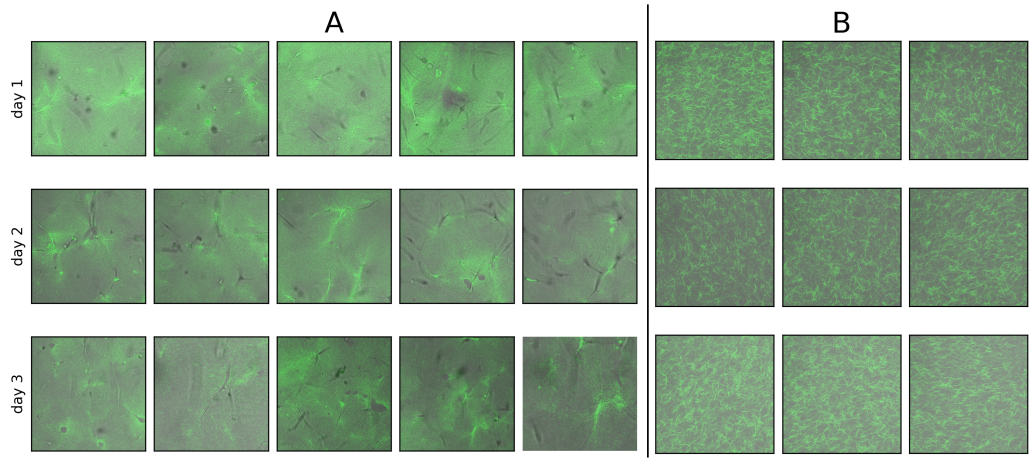

Figure S15: Imaged collagen-fiber network in respect to the cancer-associated fibroblasts. Microscopy fields of view (FOVs) on collagen fibers (reflectance) and fibroblast locations (brightfield) are overlaid. **(A)** Overlaid typical images on collagen-based 3D fibroblast cultures over incubation of 1, 2, and 3 days. For each incubation time, there are duplicate 3D culture samples. **(B)** Control collagen samples for the varied incubation times. There are differences between signal strengths depending on the imaging time for each incubation condition (day 1–3), indicating that the presence of collagen fibers rather than the strength of fibers' reflectance signal is relevant in this data. NB: All the images have been postprocessed using CLAHE. The images' width/height is 581 μm.

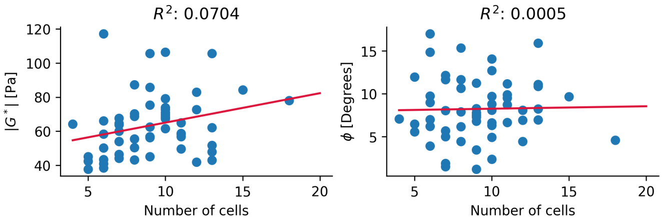

Figure S16: Estimated viscoelasticity (stiffness $|G^*|$ and phase angle ϕ) as a function of cell number. Specifically, the relation between $|G^*|$ and the cell number has a significant p-value <0.05 for the slope, but the coefficient of determination is only roughly 0.07 meaning that the number of cells predicts poorly the variation of the data. Therefore, this correlation is highly uncertain.
