# Supplementary material for: Probabilistic analysis of spatial viscoelastic cues in 3D cell culture using magnetic microrheometry
Source: Biophys J. 2024 Dec 16;124(2):351–62. doi: 10.1016/j.bpj.2024.12.010 (PMC11788488; doi:10.1016/j.bpj.2024.12.010)
Supplement: Document S2. Article plus supporting material [file mmc2.pdf]

Biophysical Journal
Article

Biophysical Society

Probabilistic analysis of spatial viscoelastic cues in 3D cell culture using magnetic microrheometry

Ossi Arasalo,[1] Arttu J. Lehtonen,[1] Mari Kielosto,[1] Markus Heinonen,[2] and Juho Pokki[1,*]
[1]Department of Electrical Engineering and Automation, Aalto University, Espoo, Finland and [2]Department of Computer Science, Aalto University, Espoo, Finland

ABSTRACT Breast tumors are typically surrounded by extracellular matrix (ECM), which is heterogeneous, not just structurally but also mechanically. Conventional rheometry is inadequate for describing cell-size-level spatial differences in ECM mechanics that are evident at micrometer scales. Optical tweezers and passive microrheometry provide a microscale resolution for the purpose but are incapable of measuring ECM viscoelasticity (the liquid-like viscous and solid-like elastic characteristics) at stiffness levels as found in breast tumor biopsies. Magnetic microrheometry records data on varying microscale viscoelasticity within 3D ECM-mimicking materials up to the biopsy-relevant stiffness. However, the measurement probe-based microrheometry data has limitations in spatial resolution. Here, we present a probabilistic modeling method—providing analysis of sparse, probe-based spatial information on microscale viscoelasticity in ECM obtained from magnetic microrheometry—in two parts. First, we validate the method's applicability for analysis of a controlled stiffness difference, based on two collagen type 1 concentrations in one sample, showing a detectable stiffness gradient in the interface of the changing concentrations. Second, we used the method to quantify and visualize differences in viscoelasticity within 3D cell cultures containing breast-cancer-associated fibroblasts, and collagen type 1 (both typically present in the tumor ECM). The fibroblasts' presence stiffens the collagen material, which aligns with previous research. Importantly, we provide probabilistic quantification of related spatial heterogeneity differences in viscoelasticity recorded by magnetic microrheometry, for the first time. The fibroblasts culturing leads to an initially higher spatial heterogeneity in the collagen stiffness. In summary, this method reports on enhanced spatial mapping of viscoelasticity in breast cancer 3D cultures, with the future potential for matching of spatial viscoelasticity distribution in the 3D cultures with the one in biopsies.

SIGNIFICANCE Breast tumor cells experience viscoelasticity, varying spatially in ECM, and these cells respond to localized cues of viscoelasticity and its spatial changes. Such ECM viscoelasticity in breast tumor 3D cell cultures for modeling breast tumor progression in biomedical applications has yet to be precisely detected. We present a probabilistic modeling method to advance magnetic microrheometry, the only current technique that can measure cell-scale viscoelasticity from the inside of cancer 3D cell cultures up to stiffness as found in breast tumor biopsies. Our method uses inherently sparse data by measurement probes of the microrheometry, and can—for the first time—quantify probabilistic spatial variability of cell-scale viscoelasticity in each microscopy field of view based on Bayesian modeling using raw measurement signals.

INTRODUCTION

Breast tumors are typically surrounded by extracellular matrix (ECM), a material that interacts with cells through mechanical and chemical cues (1). The ECM mechanics has been established as a regulator of multiple biological processes during breast tumor progression (e.g., invasion, migration, and adhesion of cancer cells) (1–3). The tumor progression often involves ECM stiffening (4), and continuous alterations of its mechanostructural properties, remodeled by cancer-associated fibroblasts (CAFs) (Fig. 1 *A*). In the typical tumor microenvironment, the ECM shows high spatial variation in mechanics at several length scales (4,5).

The spatial differences in the ECM mechanics have been studied using a variety of measurement techniques (4,6,7). Commonly, spatial information of mechanical properties has been obtained using atomic force microscopy (AFM), which can measure ECM up to the stiffness as found in

Submitted August 26, 2024, and accepted for publication December 9, 2024.
*Correspondence: juho.pokki@aalto.fi
Editor: Daphne Weihs.
https://doi.org/10.1016/j.bpj.2024.12.010
© 2024 The Author(s). Published by Elsevier Inc. on behalf of Biophysical Society.
This is an open access article under the CC BY license (http://creativecommons.org/licenses/by/4.0/).

patients at the micrometer- and submicrometer-scale resolution depending on the AFM probe size (e.g., 5 μm in (4)); however, AFM is restricted to the use of single-probe measurements at the sample's surface proximity (4). Microrheometry methods are required for multiple-probe quantification of microscale mechanics from the inside of 3D cell cultures that are cell cultures embedded in a material that mimics ECM (ECM mimics).

Microrheometry of 3D cell cultures has been carried out by existing high-resolution techniques that use a large number of small measurement probes in ECM mimics to enhance spatial resolution. These techniques, passive microrheometry (8) and active optical tweezers (9–12), can use probes with sizes from $\simeq$100 nm to $\simeq$5 μm. The techniques are used to extract ECM viscoelasticity (13), the liquid-like viscous and solid-like elastic characteristics, which regulate invasive behaviors of cells in breast cancer (1,3). To record spatially varying data on viscoelasticity, the techniques' nano- and microscale probes (14,15) enable hundreds of measurements within a single microscope's field of view (FOV). However, the use of the techniques is limited in stiffness (Young's modulus, E). They can measure only softer ECM mimics—up to E $\simeq$ 3 kPa for optical tweezers (16), and up to E $\simeq$ 10 Pa for passive microrheometry—compared with the ECM as found in breast tumor tissue biopsies (up to E = 10 kPa (4)).

Magnetic microrheometers use electromagnets for generating higher forces than optical tweezers, for viscoelasticity measurements, enabling movement of microscale magnetic probes embedded into stiffer ECM mimics in 3D cell cultures (13,17). Specifically, the recent work by Lehtonen et al. (17) demonstrates the ability of a magnetic microrheometer to operate up to the required stiffness of E = 10 kPa. In magnetic microrheometry, multiple probes can be steered simultaneously using a uniform magnetic field gradient within the field of view (18) to provide data on spatial differences, although the spatial resolution of magnetic microrheometry is limited by the generated force (i.e., a function of the field-gradient strength and the magnetization of the magnetic probes). So far, the measurements at stiffness limit of E = 10 kPa (17) have been acquired by increasing the probe magnetization using probes with sizes up to 100 μm—which decreases spatial resolution.

Increasing measurement points with smaller, micro- and submicroscale probes has allowed researchers to generate high-resolution spatial maps around cells in ECM mimics. In this way, 2D visualizations of viscoelastic and other mechanical cues, and quantification of cell-orientation-dependent behaviors, has been provided using magnetic microrheometry (up to E $\simeq$ 30 Pa) (19,20) and other microrheometry techniques (up to E $\simeq$ 3 kPa) (10–12). To date, measurements of stiffer ECM mimics for 3D cell culture have necessitated simpler analysis of variability in magnetic microrheometry with a limited spatial resolution (17). So far, magnetic microrheometry can probe ECM viscoelasticity using smaller probes sizes of $\simeq$10 μm for cell-scale data points at limited stiffness (18), theoretically, enabling increased spatial resolution at high stiffness when higher probe magnetization and larger field gradients are used (i.e., yet assumed to be experimentally shown in future).

Bayesian modeling is a powerful statistical framework for providing sample-efficient analysis of data, like the cell-scale viscoelasticity within a breast tumor-relevant ECM mimic of 3D cell culture. One prominent Bayesian technique for modeling such data in small data regime is Gaussian processes. They provide a sample efficient way for capturing nonlinear dynamics while giving probabilistic interpretability during inference (21). They have been used to model viscoelasticity data from optical tweezers (22), and strain-stress history data of variety of materials (23).

However, the spatial resolution of cell-scale viscoelasticity data (with $\simeq$10 μm probes) from magnetic microrheometry has remained underappreciated in the field due to the issue of sparsity of the data, and it is unknown whether the use of Bayesian techniques can provide robust, probabilistic information on spatial viscoelasticity, based on the data from sparsely scattered probes, and the raw measurement signals from each probe's data point.

In this article, we report about a Bayesian modeling method for magnetic microrheometry—which uses sparse data by 10-μm-diameter probes, with raw measurement signals at each data point—to quantify the spatial, microscale viscoelasticity differences from inside of 3D cell culture ECM mimics. For magnetic microrheometry, this method uses, for the first time, data of the directly measured sinusoidal magnetic probe displacements at each location to probabilistically obtain more robust estimates of the underlying heterogeneity behavior of ECM mimics (Fig. 1, *B* and *C*). Viscoelastic cues in the ECM mimic of collagen type 1—the most abundant protein in the tumor environment—were modeled as continuous fields by imposing Gaussian process priors to the unknown viscoelastic properties. Initially, we validated both the method and the microrheometry system using a precise system calibration. The actual analysis using the method was performed in two parts. First, we verified the method's performance by analyzing a controlled stiffness difference, based on two collagen type 1 concentrations in one sample. Second, we used the method to quantify viscoelasticity in 3D culture of collagen type 1 (at 1.0 mg/mL) and breast CAFs that, both, are components typically present in the tumor ECM (Fig. 1, *D–F*). To summarize, this method for cell-scale 10-μm-diameter probes has the potential to be used for precise spatial mapping of 3D cell cultures at stiffness up to E = 10 kPa, as found in breast cancer biopsies, in the case of future advancements in probe fabrication (for higher magnetization), together with advanced microrheometry (for elevated magnetic field gradients).

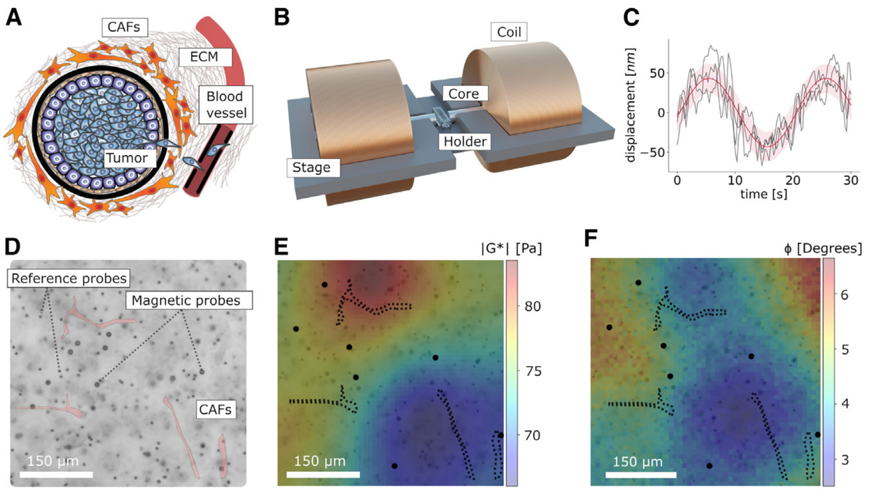

FIGURE 1 Overview of magnetic microrheometry of breast tumor tissue-relevant ECM, with data input to our Bayesian model. (*A*) In breast tumor microenvironment, a tumor is surrounded by ECM and cancer-associated fibroblast (CAF) cells within the ECM. Cancer cells from the tumor may migrate via the ECM to blood vessels to cause lethal secondary site tumors. (*B*) A magnetic microrheometer—with two electromagnets having extending cores—is used to generate oscillatory forces onto microscale magnetic probes inside a gel, an ECM mimic, or a 3D cell culture within a sample holder. The instrument is fixed on a stage enabling for mounting it on a microscope. The microrheometer allows recording data in 3D ECM mimics at multiple microscopy fields of view, simultaneously measuring several data points based on measurement probes. (*C*) Example of a probabilistic fit to measured displacement signals. A solid red line indicates the posterior mean and a shaded red region shows the 95% credible intervals. Several black lines show the signal data for one data point: subtraction between a magnetic probe and different reference probes around the magnetic probe. (*D*) Example bright-field microscopy view to reconstituted ECM of a collagen type 1 gel with the CAF cells, highlighted in red. The measurements use magnetic probes that are steered by the microrheometer's electromagnets, as well as small nonmagnetic probes (noted as reference probes) that provide reference positions for accurate displacement tracking, to omit vibrations of instrument components and the environment. (*E*) Mapping of stiffness based on absolute complex shear moduli ($|G^*|$), which were estimated from the probabilistic probe displacements fits, enabling visualization of spatial differences within a microscope field of view. (*F*) Mapping of loss factor (ϕ) in the same field of view, similarly, estimated from the probabilistic probe displacements fits.

MATERIALS AND METHODS

Preparation of calibration gels, ECM mimics, and 3D cell cultures

Measurement probes were used inside a variety of ECM mimics/gels and for recording localized viscoelasticity data using the microrheometer (Fig. 1, *B* and *C*). Specifically, each of these gels were prepared using two types of spherical probes: magnetic probes (nominal diameter of 10 μm; Sigma-Aldrich, St. Louis, MO, 49664) and nonmagnetic probes (Polysciences, Warrington, PA, 24293) in aqueous suspensions (Fig. 1 *D*). The probes were pipetted from each probe batch's (bottle's) bottom part after mixing the batch. The probes were introduced into the gels by mixing them into the medium constituent of the gels, except for silicone oil. To avoid introduction of nonsolving water within silicone oil, we used both probes in a dry form, resuspended in the oil.

There were three types of ECM mimics/gels, measured by microrheometry, placed in polydimethylsiloxane sample holders with a glass bottom ($20 \times 4.5 \times 3.3$ mm^3), and each of the three types had a specific preparation. First, a silicone oil gel was used simply as it is (Sigma, 63148-62-9, $\mu = 30{,}000$ cSt), together with the dried probes mixed in the oil. Second, an ECM mimic of collagen gel consisted of two concentrations, 1 and 2 mg/mL, of rat-tail collagen type 1 (Fisher Scientific, Corning, Waltham, MA, 354236 and high-concentration collagen type 1, Corning 354249) aliquots, which were simultaneously prepared into each sample holder. In both concentrations, the spherical magnetic and nonmagnetic probes were diluted to make up the final volume fractions of 0.06 and 0.03%, respectively. Each sample holder was separated with a glass slide (Sigma-Aldrich, BR470055) into two wells, each of which contained 400 μL of each aliquot (i.e., collagen with concentrations of 1 or 2 mg/mL). The sample was let to polymerize in 37°C for 1 h. The glass slide was removed and the sample was measured. Third, we prepared 3D gels of collagen and human breast CAFs, which were obtained from Pelobiotech (Planegg, Germany, PB-CH-459-6411) and used within 8 passages after arrival. The CAFs were cultured in fibroblast basal medium containing growth supplements such as 0.03 ng/mL human TGF-β1 and 2% fetal calf serum, all from Pelobiotech (PB-BH-400-0090 and PB-MH-400-9099). The cells were maintained at 37°C under 5% CO_2. To prepare the 3D CAF culture samples for microrheometry experiments, the aqueous solutions of the magnetic and nonmagnetic probes were both introduced as dilutions of 0.05% to the medium of 1× Dulbecco's modified Eagle's medium (DMEM) (Gibco, 41965062; Thermo Fisher Scientific) containing 1% penicillin-streptomycin (Gibco, 15140122). For passaging, the CAFs were then treated with 0.05% trypsin/EDTA (Gibco, 25300062) to detach them, centrifuged for 5 min and diluted to a seeding density of 2.4×10^5 cells/mL in 1× DMEM containing both probe types (magnetic and nonmagnetic). After that, we neutralized the collagen ECM product (as used for the collagen gels). For the neutralization, the volume ratio between 10× DMEM (Gibco, 12100061) containing $NaHCO_3$ (Sigma-Aldrich, S5761) and collagen was 1:9, and they were mixed by shaking. Finally, the mixture of 1× DMEM, the magnetic and nonmagnetic (reference) probes, as well as CAFs were thoroughly mixed on ice with the neutralized collagen with a final concentration of 1 mg/mL. This mixture was divided into custom-made sample holders. Control samples were made in the same way, but without the CAF cells. All these collagen dilutions (with and without the CAF cells) were allowed to gelate in an incubator at 37°C for 40 min before adding the CAF growth medium. The measurements were done after 24 h (day 1), 48 h (day 2), and 72 h (day 3) using the magnetic microrheometer. The cell viability was quantified after 24 h of incubation using the Cyto3D Live-Dead Assay Kit (TheWell Bioscience, North Brunswick, NJ) according to the manufacturer's instructions (see supporting material, section 1.1). The 3D CAF culture and control samples were prepared for collagen fiber imaging in the same way as described above, except without the magnetic and nonmagnetic probes, μ-Slide 18 Wells (Ibidi, Gräfelfing, Germany, 81817) were used instead of holders. Reflectance microscopy images of collagen fibers were acquired with an inverted confocal microscope using a 633 nm laser (Leica, Wetzlar, Germany, TCS SP8).

Setup of magnetic microrheometry to record viscoelasticity data from inside of 3D ECM mimics

The used magnetic probe-based microrheometer is based on a system proposed in (18) and advanced in (17), in respect to capabilities for

multiple-probe tracking, to reach a Young's modulus of 10 kPa and to quantify heterogeneity within tumor-relevant 3D cell culture ECM mimics/gels (Fig. 1). This microrheometer is composed of a stage for mounting it on a microscope, and two custom-wound electromagnets with Co-Fe cores (Vacoflux 50, Vacuumschmelze, Hanau, Germany), which enable variation of the following parameters separately: a magnetic field that magnetizes magnetic probes and a magnetic field gradient that exerts forces on the magnetized probes within the ECM mimic/gel inside the sample holder (Fig. 1 *A*).

In short, the microrheometer operates by exerting oscillatory magnetic forces on spherical magnetic probes within the ECM mimic/gel in each sample holder (Fig. 1, *B* and *C*), and detecting the probe displacements (Fig. 1 *D*) to compute the viscoelasticity data (Fig. 1, *E* and *F*) (17). Specifically, we induced time (t)-dependent sinusoidal forces (with an amplitude of 0.3 nN at a controlled frequency, f, of 0.05 Hz) onto each magnetic probe (numbered i), which provided a sinusoidal displacement response with an amplitude of 25–140 nm (Eq. 1 and Fig. 1 *D*). The displacement tracking of the probes was performed using a microscope camera, with a pixel size of 6.5 μm (Hamamatsu Orca Flash 4.0, Hamamatsu, Japan) attached to the microscope (Zeiss Axiovert 200M, Oberkochen, Germany) with a 20× objective. The objective's numerical aperture is 0.3, and it relates to a resolution of 0.8 μm based on Abbe's diffraction limit when green light at a wavelength of 0.5 μm is assumed. This 20× objective results in a camera pixel denoting 0.325 μm in the sample. The force (F_i) and displacement (d_i) data at the linear viscoelasticity regime were used to compute viscoelasticity around each (i^{th}) magnetic probe, with a radius of r_i. As the viscoelasticity parameters, the absolute complex shear modulus ($|G^*|$), which indicates a material stiffness, and the phase angle (ϕ), which describes the proportion between the material's viscous and elastic characteristics (24), were computed according to the following equation (for each magnetic probe displacement):

$$d_i(t) = \frac{2 f_v r_i^2}{9|G^*|} sin(2\pi f t - \phi), \tag{1}$$

where f_v is the volumetric force calibration value assuming a constant magnetization of the magnetic probes (i.e., relating to force, F_i, normalized by the total volume of each probe: $f_v = F_i / \left(4/3 \pi r_i^3\right)$). Details of the use of Eq. 1 in the computation is described in the next section on approach to analyzing spatial viscoelasticity in 3D ECM mimics based on Bayesian modeling. Similarly, details for extracting the volumetric force are described in a further materials and methods section on system calibration. Further information about deriving Eq. 1 can be found in supporting material, section 1.2.

We extracted each magnetic probe displacements ($d_i(t)$) in a process where we subtracted the nonmagnetic probe movement from the raw displacement data of each magnetic probe, to remove the noise in the magnetic probe data (i.e., due to the microscope's magnetic objective's movement while applying the magnetic field gradients, as well as vibrations of the microscope stage (17,18)). The maximum displacement of three closest reference probes from the magnetic probe was used as the nonmagnetic probe's movement in the subtraction. The magnetic probe-reference probe distances were limited to span between 50 and 200 μm, because we observed that the reference-probe movement varies spatially. Fig. 1 *D* shows exemplary displacement signal data for a data point, specifically, the data point is obtained via subtraction between a magnetic probe's raw displacements (with noise) and a reference probe's displacements (capturing the noise). For the data point in Fig. 1 *D*, there are three displacement signals, because this subtraction has been performed for the three closest reference probes.

We captured a video feed of a microscope field of view (FOV) with multiple moving magnetic probes (surrounded by the reference probes) using a rate of 25 ms that corresponds to 0.5° in phase angles, accounted for in the phase-angle estimation. After synchronizing each data set on forces exerted on the probes, with the related data set on the probes displacements, we subsampled the aligned data sets to one-tenth of the original data length for computational time saving.

Approach to analyzing spatial viscoelasticity in 3D ECM mimics based on Bayesian modeling

We have developed a Bayesian modeling method (Fig. 2) to extract spatial information from the sparse viscoelasticity data by the magnetic microrheometer (Fig. 1, *E* and *F*). While the magnetic microrheometer is advantageous in measuring the oscillatory microrheology of stiff ECM mimics up to Young's moduli of 10 kPa (17), it has a reduced spatial resolution compared with other microrheometry techniques. Equation 1 shows that larger magnetic probes are required for detecting a displacement signal from stiffer gels for 3D cell culture. In this scenario, the number of unique measurement points within a single FOV is often limited by the count of the larger magnetic probes at increased concentrations, when measuring stiffer materials. Furthermore, the maximal concentration of the probes is constrained by the requirement of no interactions between the probes (Eq. 1), denoting that the concentration has to be carefully adjusted, while maximizing the number of data points. For a sanity check, we tested our adjusted method of dispersing magnetic probes in the samples, described in the section preparation of calibration gels, ECM mimics and 3D cell cultures. This test screened for evidence of problematic clustering or aggregation of the probes, by calculating the Ripley's G-function based on our data (25). The G-function values, depending on probe-to-probe distances, was then compared with the theoretical homogeneous point process (Fig. S1). The lack of noticeable differences in the comparison indicate the lack of the undesired clustering/aggregation. The data in current magnetic microrheometry are sparse, and the signals have also an inherent noise. To tackle these challenges, we have designed a Bayesian model to capture the underlying viscoelastic properties of the ECM mimics with an improved spatial information compared with raw measurements.

Viscoelastic properties of the 3D cell culture gels are estimated using Bayesian modeling, also illustrated in Fig. 2. Now, instead of assuming viscoelasticity data ($|G^*|$ and ϕ) to be point-like values as in Eq. 1, we have imposed Gaussian process priors on the viscoelastic parameters, and model them as spatially varying fields (Fig. 1, *E* and *F*), as specifically described in the following equations:

$$d_i(t) \sim \text{Student's t}\left(\nu, \frac{2 f_v r_i^{*2}}{9 g(x_i)} sin(2\pi f t - h(x_i)), \sigma_i\right) \tag{2}$$

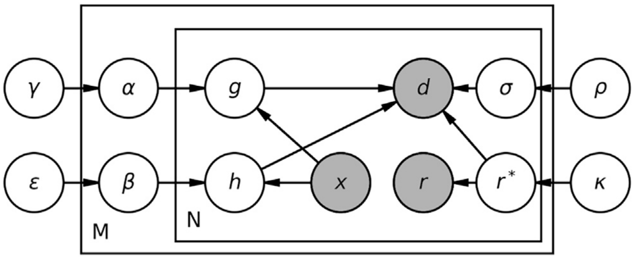

FIGURE 2 Plate diagram of the proposed probabilistic model for probing ECM mimic's viscoelasticity and its heterogeneity in 3D cell culture. Gray circles represent the observed quantities and white circles show the modeled random variables. The model works for a number of N magnetic probes measuring in M different microscopy fields of view. Specifically, the variables d, x, and r are the measured probe displacements, locations, and radii values, respectively. The characters g (for stiffness) and h (for the phase angle) are the Gaussian process priors for the viscoelastic fields. Furthermore, α and β are the hyperpriors controlling the shape of the Gaussian processes at the field of view level, and γ and ϵ the global parameters. Then, σ is the modeled displacement signal noise separate for each measured signal constrained with global hyperpriors (ρ). Finally, probes radius is estimated with a measurement error model r^* constrained by the the real probe measurements and hyperpriors (κ).

$$g(x) \sim \text{Softplus}\left(\mathcal{GP}\left(0, K_g(x, x')\right) + \mu_{|G|*}\right) \tag{3}$$

$$h(x) \sim \arcsin\left(\left(\text{sigmoid}\left(\mathcal{GP}(0, K_h(x, x')) + \mu_{\phi}\right)\right)\right) \tag{4}$$

Equation 2 models each magnetic probe displacement signal (d_i) with a Student's t distribution, serving as a likelihood function. Longer tails of such likelihood function enable to handle noisy displacement signals that are subject to random outliers due to the use of a centroid-based probe tracker (17) (i.e., switching between adjacent pixels). The variable r_i^* is the estimated true magnetic probe size. We assume an inverse gamma prior, noted as ν. Furthermore, the measurements' noise is modeled by σ_i, as we have employed a hierarchical prior that enables to handle magnetic-probe-specific noise contributions. Specifically, different levels of noise exist in the captured image sequence due to: the thickness of the material with optical effects, some probes being out-of-focus, and the cell-related localized dynamic alterations within the ECM mimic. Thus, we allowed different levels of signal noise for each individual probe but use partial hierarchical pooling to improve parameter estimation (shown in supporting material, section 1.3).

Equation 3 (for $g(x)$) and 4 (for $h(x)$) are Gaussian processes that model continuous viscoelastic fields of the absolute complex shear modulus ($|G^*|$), and the phase angle (ϕ), respectively. Furthermore, both $g(x_i)$ and $h(x_i)$ use the magnetic probe coordinates (x) as inputs for $|G^*|$ and ϕ, respectively. We constrained the Gaussian processes' outputs to physically feasible regions. Specifically, $g(x_i)$ is set to be positive with a Softplus function, and $h(x_i)$ is restricted to generate values between $[0, \pi/2]$. Both $g(x_i)$ and $h(x_i))$ use the parameters, $\mu_{|G*|}$ and μ_{ϕ}, respectively, to capture the mean variation behavior in the processes. These parameters are unique for each measured FOV and have hierarchical priors (details found in supporting material, section 1.3). Specifically, we have added these two averaging terms ($\mu_{|G*|}$ and μ_{ϕ}) to extrapolate as realistic values as possible based on the data. The hyperprior for the mean has to be chosen roughly informatively to prevent nonidentifiability. For $|G^*|$, a justified choice for our experiments is $\mathcal{N}(50, 15)$. For ϕ, a justified choice is $\mathcal{N}(0, 0.3)$ in the transformed space of arcsin(sigmoid(x)), where $x \in \mathbb{R}$, because we are measuring only ECM mimics that exhibit characteristics of viscoelastic solids with the expected ϕ mean close to 0 rad. Similarly, for the case of ECM mimics with mostly viscous-like characteristics, we would assume the expected mean to be close to $\pi/2$ rad of purely viscous material; thus, the prior would be $\mathcal{N}(\pi/2, 0.3)$.

The spatial variability of the measured ECM mimics is assumed to be smooth, therefore, we have chosen covariance functions (K_g in Eq. 3, and K_h in Eq. 4), which are exponentiated quadratic functions with white noise. The parameters controlling the shape of the functions are further constrained hierarchically to be similar across multiple measured FOVs because collagen matrices are known to possess large variations between different length scales. Thus, the covariance functions are defined in the following equations, where the function K is used for the case of K_g, as well as K_h:

$$K(x, x') = \alpha_j^2 \exp\left(-\frac{|x - x'|^2}{2l_j^2}\right) + \sigma_\sigma^2 \mathbf{I}_n \tag{5}$$

$$K_g : \alpha_\mu \sim \text{Student's t } (3, 0, 20) \tag{6}$$

$$K_g : \alpha_\sigma \sim \text{Half} - \mathcal{N}(0, 5) \tag{7}$$

$$K_h : \alpha_\mu \sim \mathcal{N}(0, 5) \tag{8}$$

$$K_h : \alpha_\sigma \sim \text{Half} - \mathcal{N}(0, 1) \tag{9}$$

$$l_\sigma, \sigma_\sigma \sim \text{Half} - \mathcal{N}(0, 1) \tag{10}$$

$$\alpha_j \sim \text{Softplus}\left(\mathcal{N}\left(\alpha_\mu, \alpha_\sigma\right)\right) \tag{11}$$

$$l_j \sim \text{GIG}(p = 2, a = 15, b = l_\sigma) \tag{12}$$

Equation 5 is the covariance as a function of the following variables: x and x' are the magnetic probe locations, α_j is an amplitude, σ_σ is the noise level, and l_j is the length scale separate for each dimension. $\mathbf{I}_n$ denotes for the identity matrix. Equations 6, 7, 8, 9, 10, and 11 show noncentered parameterization that is used for the hierarchical amplitude prior. The variable α_μ can be interpreted as the condition/material specific mean for ECM mimic's heterogeneity. Similarly α_σ indicates the variability between measured FOV heterogeneity values. If we assume minimal errors caused by the experimental design (between FOVs), α_σ can be seen as a measure of the locality of ECM mimics heterogeneity. In other words, it captures how similarly the material varies when looking at small (hundreds of micrometers) subsets of the full millimeter-scale material. The scale of these priors has to be sufficiently large to capture the level of the heterogeneity in the ECM mimics, based on the prior domain expertise about the expected behavior. The evaluation and the choice of priors is explained in the sensitivity analysis of the supporting material, section 2.1. Equation 12 defines the length scale (l_j) that follows the generalized inverse Gaussian distribution that is a zero boundary avoiding prior, simultaneously avoiding excessive values (converging to flat surfaces) (26). Joint hyperprior l_σ is used to constrain different length scale parameters to be similar across different measured FOVs as they are measuring the same ECM mimic.

Furthermore, the accuracy of the magnetic probe radius estimate is crucial to providing accurate data on viscoelasticity. The probe radius estimate has a squared dependence on the probe displacement (Eq. 1). Therefore, this radius has been modeled with a measurement error model. In the model, the true quantities are considered as missing data, and we have a measurement for each missing data point with a known error. Thus, the true quantities have been modeled using a random variable and they have been inferred among other parameters. The model for the probe radius (r_i) is as follows:

$$r_\mu \sim \mathcal{N}(6, 1) \tag{13}$$

$$r_\sigma \sim \text{Inverse Gamma}(\alpha = 2, \beta = 0.5) \tag{14}$$

$$r_i^* \sim \mathcal{N}\left(r_\mu, r_\sigma\right) \tag{15}$$

$$r_i \sim \mathcal{N}\left(r_i^*, \tau\right) \tag{16}$$

where r_μ and r_σ are the hyperpriors for the probe radius. The character r^* is the estimated true magnetic probe size and τ is a numerical value of the estimated measurement error. In our experiments we use $\tau = 0.1$, which corresponds approximately to ± 1 pixel of error in the radius estimation. With this modeling choice, some of the uncertainty in the data is now explained by the noise in the radius estimate affecting the final viscoelasticity fields, defined in Eqs. 3 and 4. However, these field-related priors and τ have to be defined carefully to avoid excessive flexibility, as the model can now explain the variations in the amplitude by either spatially varying viscoelasticity fields (that is of interest), or by recalibrating poorly estimated radius values (that is to be avoided).

Finally, the volumetric force calibration constant (f_v) is also modeled as a random variable and its estimation is described in the next materials and methods section on system calibration. The subsequently inferred distribution is randomly sampled during the model fitting to include the possible uncertainty in the calibration constant.

The probabilistic modeling is implemented by the Stan programming language (version 2.28) and sampled using the build-in MCMC sampler (27). We have run the sampler with 4 parallel chains with 1000 samples in each chain. The quality of the samples was verified using the build-in diagnostics. The sensitivity analyses to choose the priors are described in the supporting material, section 2.1.

System calibration

The microrheometer was calibrated before carrying out the experiments. We have followed an established calibration procedure (13,24), where a purely viscous silicone oil gel is prepared and manipulated with multiple magnetic probes. This gel's mechanical properties, specifically viscosity values, are known, therefore, we have applied the Stokes law to infer the volumetric force calibration constant (f_v), as in the following equations:

$$6\pi r\eta \frac{dx}{dt} = MV\nabla B \tag{17}$$

$$6\pi r\eta \frac{dx}{dt} = MV\underbrace{\nabla B_0 \sin(\omega t)}_{\nabla B} \tag{18}$$

$$x(t) = -\frac{2}{9\eta\omega} r^2 \underbrace{M\nabla B_0}_{f_v} \cos(\omega t) + C \tag{19}$$

Equation 17 shows the equality of the Stokes drag force with the magnetic force in the equilibrium. Here, η is the dynamic viscosity of the silicone oil gel at room temperature, M and V are the magnetization and volume of the magnetic probes, respectively. The magnetic field gradient (∇B) was estimated as a sinusoid function, parallel to the axis between the coils, with an amplitude of ∇B_0 (Eq. 18). In practice, ∇B_0 varies spatially within the microscopes field of view, because the magnetic field is not perfectly homogeneous. However, based on COMSOL simulations, the variation is ⟨0.5% meaning that the error caused by a single global parameter instead of a spatially varying field will be small compared with other sources of error (17). As our goal of keeping the model uncertainty aware, these parameters have been estimated using Bayesian modeling. Similarly, as for the previously introduced spatial model, uncertain magnetic probe radii have been corrected with a measurement error model (Eqs. 13, 14, 15, and 16). The probe magnetization M and the magnetic field gradient amplitude (∇B_0) have been fused together into the volumetric force calibration constant (f_v) possessing a prior of $\mathcal{N}(280000, 14000.0)$. The choice is largely arbitrary, but it generates realistic signals evident in the prior predictive distribution (Fig. S6). As an alternative solution, it would be possible to model these parameters separately and use informative priors from COMSOL simulations and measured magnetic probe magnetization curves. However, multiplicative degeneracy between the parameters would complicate the interpretation of the posterior fits and further these parameters are of no particular relevance for the spatial model.

Equation 19 shows the final relation used to fit the raw displacement data for our modeling. For the f_v estimation, the likelihood function has been chosen as a Gaussian distribution with a common standard deviation for each signal. In contrast to the spatial model, the displacements of the magnetic probes are larger in the silicone oil gel, resulting in smaller errors in the tracking algorithm. Thus, the random noise is in practice the same across different measured signals. Small errors in signal synchronization and drifting of the probes have also been corrected. The full modeling details can be found in supporting material, section 1.4. The MCMC sampling was implemented in the Tensorflow probability (version 0.19.0) using a NUTS sampler (28). The sampler was initialized with a MAP estimate. We run 4 parallel chains with 2000 adaptation steps, then 2000 burn ins, and finally 2000 posterior samples (example fits shown in Fig. S7).

RESULTS

Initially, we report the system calibration results of the modeling method and the microrheometry. The subsequent results about the data-based modeling are presented in the following two parts, specifically, on the method's validation, verification by resolving a stiffness difference, and the application in quantifying viscoelasticity and its heterogeneity using breast CAFs in 3D collagen as 3D culture of interest.

System calibration

Our data incorporating model ($n = 40$ probes) shows a predictive advantage with coefficient of variation dropping below one-tenth of the raw data results, while the model provides a volumetric force calibration constant that closely matches with the constant based on the data alone (Table 1).

Next, we studied the parameters, to which viscoelasticity results are sensitive. For the absolute complex shear modulus, the most influential parameter is the probe radius, to which the model estimates an error of $\simeq 0.78$ pixels, or 0.25 μm. This is in line with our imaging resolution having pixel size of 0.325 μm.

For the phase angle, the most influential parameter is the time delay between the two sinusoidal data sets: each magnetic probe displacements (in an ECM mimic or a gel), and the forces experienced by the same probe. We estimated the error in time synchronization from the phase shifts in the sinusoidal fits. The mean phase shift uncertainty estimated by the model is $\simeq 2.8^\circ$. This is higher than the theoretical limit of 0.5° from temporal imaging resolution and may be due to spatially varying bulk flow caused by magnetic probes (i.e., specific to the use of silicone oil for calibration).

Verification by resolving a stiffness difference

For verification, we used the probabilistic model to analyze relevant mechanical data from the collagen gels that have an artificially generated difference in stiffness (i.e., absolute complex shear modulus $|G^*|$). Fig. 3, A and B show the posterior means of two exemplary fields of view (FOVs) for the collagen stiffness values, demonstrating that the model provides a sufficient resolution to resolve the spatial difference in stiffness. Specifically, a stiffness gradient was successfully detected based on 7 and 4 probe-based measurements, in the interface between the collagen concentrations, shown in Fig. 3, A and B, respectively.

TABLE 1 Estimate of calibration constant for volumetric force (f_v) shows an order of magnitude lower coefficient of variation for our modeling method compared with raw data results

	Mean (N/m^3)	SD (N/m^3)	CV (%)
Data	317,616.3	36,712.1	11.6
Model	316,039.3	2573.0	0.8

Fig. 3, *C* and *D* show the corresponding spatial changes in stiffness evaluated along the on-axis dimension (x) where the gradient was artificially created, whereas there are only random stiffness variations along the off-axis dimension perpendicular to the artificial gradient (Figs. 3, *A*, *B*, and S8). Our spatial analysis of the collagen gels shows that the concentration of 2 mg/mL expectedly provides approximately double the stiffness compared with the concentration of 1 mg/mL. Testing a further refined resolution of the model is unfeasible using these gels, because the gels lack measurement probes in the zone separating the two different collagen concentrations (*gray region* in Fig. 3, *C* and *D*). Despite this, our model estimates a realistic stiffness gradient around the measurement data, in the interface between the collagen concentrations, representing the artificially created stiffness difference. Besides, our model provides better calibrated uncertainty estimates compared with the conventional analysis pipelines using raw data including also potential biases and uncertainty. Now, the uncertainties in the probe radii estimation and the fitting to the sinusoidal displacement signals, among other factors, contribute to the credible intervals of the Gaussian process. In comparison, the conventional analysis pipelines do not account for these uncertainty factors in a principled way, which may lead to both overconfidence and sensitivity to outliers, in the percentile intervals, visible in Fig. 3, *C* and *D*.

For further model validation, we found that the probabilistic estimates provide the same magnitude as estimates calculated from the deterministic pipeline (Fig. S9, *A* and *B*). The proportion of large deviations from the raw measurements is also relatively low and is mostly within 10 Pa or 2.5° (Fig. S9, *D* and *E*). The raw measurements incorporate noise and are prone to outliers due to small measured amplitudes. The magnetic probe displacements, obtained using subtractions between each magnetic probe and the surrounding reference probes, can vary largely for a single magnetic probe, which may result in considerably high uncertainty. However, our method is robust to such variations, as shown in Fig. S10.

The magnetic probe manufacturer reports the probes' nominal radius to be 5.24 μm, with a 0.05 μm (0.02 pixels) standard deviation. Our modeling method estimates the average probe radius (r_μ) as 6.14 μm, having a 0.36 μm standard deviation (r_σ). This estimation for the average probe radii provides an elevated value compared with the one reported by the manufacturer, explainable by batch-to-batch variation, and an existing variation within each probe

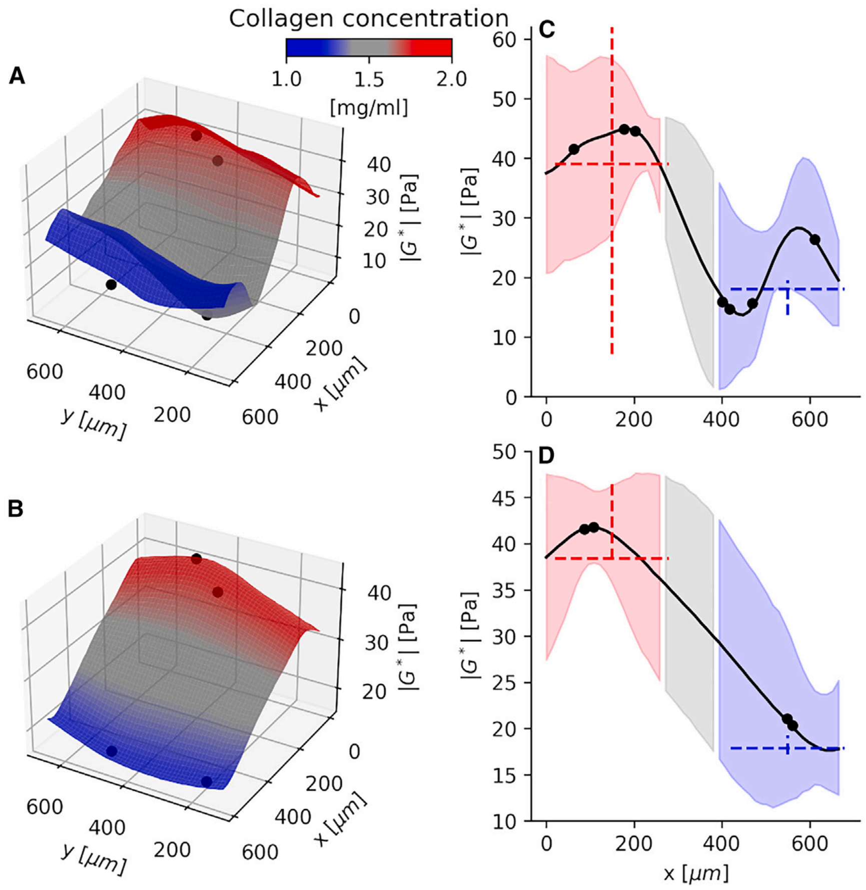

FIGURE 3 Stiffness gradients detected in the interface of two collagen concentrations, known to change the stiffness level. (*A* and *B*) The model's posterior means for the stiffness ($|G^*|$) fields, as a function of the planar coordinates, *x* and *y*. (*C* and *D*) The changing stiffness along the *x* axis, perpendicular to the borderline between the two collagen concentrations. The values are extracted at the centerpoint of *y* axis and at the FOV's center. Shaded regions indicate the 95% credible intervals. Red and blue colors correspond roughly to the regions with 2.0 and 1.0 mg/mL of the collagen, respectively, and the gray color indicates the position where the glass cover slide was positioned and where the concentration (and stiffness) is expected to be between the two concentrations. Black dots are the projected measurement points. Dashed lines describe the raw data from each collagen concentration: the median level, and the range of values between the 5th and 95th percentiles.

batch, as we pipette probes in each batch's bottom part after mixing the batch, potentially capturing the slightly heavier (and larger) probes (i.e., a part of our experimental method). A similar difference was also observed in (18), which used direct microscopy measurements of the probes. The systematic increase does not affect the stiffness (the absolute complex shear modulus) as the same difference is present during the system calibration.

Furthermore, an inclusion of a measurement error model to the radius estimate gives near identical results for the absolute complex shear modulus (−0.14 Pa difference on average), validating that the model does not scale radius values unrealistically. Specifically, the adjusted probe radius values are extremely conservative, as is evident in Figs. S9 *C* and S11. This more comprehensive treatment of the known sources of uncertainty results in a $\simeq 34\%$ increased standard deviation in the spatial viscoelasticity field. The radius estimation has uncertainty, and—due to the quadratic scaling—even small changes can have a considerable impact on the final stiffness value (absolute complex shear modulus). Thus, by adding the measurement error model, the key inaccuracies of the method (related to the probe radius's standard deviation) have been captured in a principled way.

Breast CAFs in 3D collagen as 3D culture of interest

Next, our modeling method was used to analyze collagen viscoelasticity in 3D CAF cell culture (Fig. 4), mimicking the breast tumor microenvironment (Fig. 1 *A*). Our analysis used two types of samples, 3D cultures (with the CAFs embedded in 3D collagen) and respective controls (with pure collagen), both measured over 3 days of incubation. Fig. 4 *A* shows the posterior means of all measurement probes, within three separate samples at every incubation time (all probes $n = 848$ consisting of an average of 7 probes/FOV, with further details noted in Table S1). We evaluated statistical comparisons by visualizing the distribution data and reporting the exact probabilities. A cutoff of 95% was used when claiming a difference or an effect in the comparisons. Shapes of the distributions provide valuable statistical information; thus, we are not limiting the analyses only to the cutoff. It is evident from the obtained spatial information that particularly stiffness shows high variability (width of the distributions) within the 3D cultures, as shown in Figs. 4, *A*–*C*, S12, and S13. Thus, it is necessary to separate the analysis between the one for the ECM mimic's viscoelasticity and the one for the variability factors.

We initially extracted collagen viscoelasticity by estimating the population-level means (Fig. 4 *D*). For this information on the collagen viscoelasticity, we have removed the overall collagen heterogeneity (Fig. 4 *B*) and the variability between microscopy FOVs (Fig. 4 *C*), as defined by the model ($\mu_{|G^*|}$ in Eq. 3, and μ_ϕ in Eq. 4). Our analysis shows that the presence of CAFs initially stiffens the 3D collagen material (Fig. 4 *D*), which is in line with the previous literature on fibroblasts often stiffening collagen material and contracting it (18,29,30). Specifically, the CAFs stiffen the collagen material at incubations of 1 and 2 days. At the incubation of 3 days, we also confirmed stiffening in one 3D CAF culture repetition, but the results for the two other repetitions are inconclusive (Table S2). We lack evidence on significant alterations in the collagen stiffness due to CAFs over time considering the incubations of 1–3 days (Table S3). This collagen stiffening is consistent with the detected elastic collagen fibers, generated between CAFs, and there is a lack of obvious changes in the fibers network between the incubation times (Figs. S14 and S15).

Then, changes in the collagen phase angle due to CAFs are reported, which have previously been unstudied (Fig. 4 *D*). We found that the phase angles decrease in some of the 3D CAF culture samples, but the decreases are inconclusive within the entire population of sample repetitions (Table S2). The decreases in the collagen phase angles denote an increased elastic-like behavior, likely relating to the elastic collagen fibers between the CAFs (Fig. S15). We lack evidence on significant time-dependent alterations in the collagen phase angle due to CAFs, as well as changes in the structural basis, the elastic collagen fiber network (Table S3 and Fig. S15). These population-level means (Fig. 4 *D*) indicate weaker differences in the phase angles between the 3D cultures and the controls, in comparison with the full posterior (Fig. 4 *A*), with biases due to the variability factors that likely falsely indicate stronger differences (Fig. 4, *B* and *C*).

We further analyzed the variability factors, particularly spatial variations and heterogeneity generated by CAFs in the collagen-based 3D CAF cultures (Fig. 4, *B* and *C*). Initially, we considered the collagen spatial heterogeneity on the entire collagen material's level. For the purpose, we examined the difference: $\alpha_\mu^{\mathrm{CAF}} - \alpha_\mu^{\mathrm{control}}$. Considering this overall heterogeneity combining all FOV data together, there is an initial increased heterogeneity in stiffness due to CAFs, at the incubation of 1 day (Fig. 4 *B*). A maintenance of the elevated heterogeneity is suggested with the noted probabilities during incubation over 2 days (93%) and 3 days (73%). In addition, there is a weaker indication of decreasing heterogeneity in the phase angle due to CAFs, based on distribution shapes and probabilities from 25 to 43% (Fig. 4 *B*). Overall, the increased initial heterogeneity in stiffness is supported by the presence of heterogeneously distributed, elastic collagen fiber structures in 3D CAF cultures (Figs. S14 and S15).

Next, we refined the analysis to quantify heterogeneity differences between FOVs based on the parameter: α_σ (Fig. 4 *C*). The results suggest that the heterogeneity in stiffness has variation between the measured FOVs as a result of initial 3D CAF culturing over 1 day, whereas there is a weak indication of simultaneous reduction of the heterogeneity differences between the FOVs in the phase angle (Fig. 4 *C*).

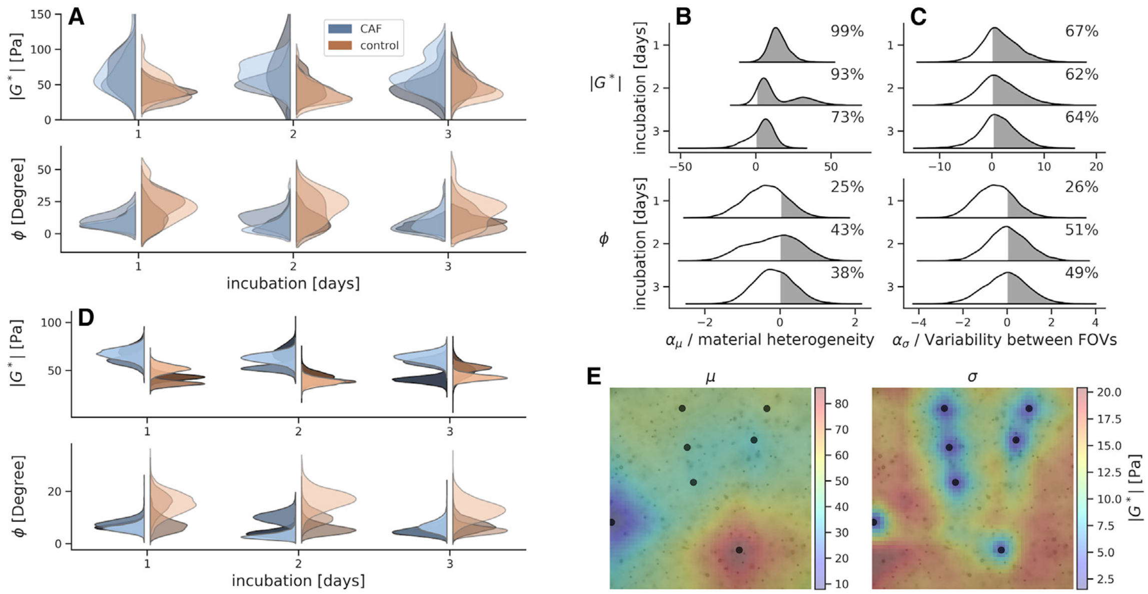

FIGURE 4 Method used for analysis of viscoelasticity in collagen material remodeled by the CAFs. (*A*) Posterior means of all the individual magnetic probes at different incubation times for both the stiffness ($|G^*|$) and the phase angle (ϕ) are shown. The graphs are based on Eq. 3 for stiffness and Eq. 4 for the phase angle. The different shades of blue and red colors represent the three different experimental samples, with 848 probes altogether. (*B*) Posterior distributions representing the difference between the heterogeneity observed in 3D CAF cell culture samples and the corresponding control collagen. The estimated difference in average heterogeneity of ECM mimic samples for stiffness and the phase angle (i.e., $\alpha_\mu^{\text{CAF}} - \alpha_\mu^{\text{control}}$). The values together with the shaded regions show the probability of 3D CAF cell cultures having higher stiffness than the control samples. (*C*) Posterior distributions representing the difference in variability between FOVs, which gives an indication of the *locality* of the variations in the ECM mimic in 3D CAF cell cultures. (*D*) Estimated population level means viscoelastic parameters, stiffness ($\mu_{|G^*|}$), and the phase angle (μ_ϕ). (*E*) Visualization of an examplary spatial field of stiffness ($|G^*|$) estimated with the proposed model. The character μ indicates the posterior mean and σ notes the posterior standard deviation/uncertainty. The black dots are the measurement points (i.e., probes for the microrheometry).

The results can be interpreted that the CAFs are responsible for generating localized areas that can change the overall heterogeneity in viscoelasticity, and there are likely areas of higher and lower heterogeneity on the millimeter scale in the 3D cultures (i.e., structural comparison in Figs. S14 and S15). These changes in the 3D cultures are not only highly localized but also varied, likely depending on the specific CAF cell, which is in line with the heterogeneous behaviors known to exist within the CAF population (31).

Lastly, we showed that our model can be used for visualization of the spatially varying properties in the ECM mimic of the collagen material. Fig. 4 *E* shows a representative example mapping of an inferred stiffness ($|G^*|$) in a 3D CAF cell culture sample. The visualization shows the posterior mean (μ) and the standard deviation (uncertainty of the estimate, σ). Interestingly, the stiffness varies by approximately an order of magnitude within the measured FOV. Expectedly, the uncertainty is lowest at the measured magnetic probes locations, and increases rapidly in the extrapolation regions of the model.

DISCUSSION

Overall, we have presented a method with a proven ability to quantify spatial viscoelastic differences and gradients within ECM mimics of 3D cell culture models that has a wide range of use cases from understanding cell migration mechanisms (32,33) to tumor progression (3). Applying our spatial Bayesian model to magnetic microrheometry data shows that we can obtain realistic stiffness gradient estimates with only a handful of magnetic probes, making this method likely applicable to measurements by other techniques (e.g., optical tweezers and AFM). While the currently limited number of probes in magnetic microrheometry has its downsides in a reduced spatial resolution, the microrheometry is beneficial for use in cancer 3D cell cultures up to the stiffness as found in breast cancer biopsies (17,34,35), and it minimizes the contribution of the probes to the measurements and allows to gather information about the spatial dynamics as well as force exertion (18,36). The further specific discussion paragraphs are on the aspects of the method development, its validation and its applications.

Considering how the method uses the magnetic and reference probes, we utilized the Gaussian process priors to provide useful regularization to the probes displacement fits that are behind the data points in each microscopy FOV. This is the case, especially for magnetic probes that have less than three reference probes within the predefined acceptance range (based on magnetic probe-reference probe distance). The model's behavior is averaging but still allows the possibility of extracting the physical, high spatial variations as it is evident in Fig. 4 *E*. The probe displacement

signals with higher uncertainty remain to be still approximated (with a higher uncertainty shown) with incorporation to the final analysis and improvement of the data efficiency, important in the sparse data regime.

A more precise system calibration, reaching to approximately one tenth in coefficient of variation compared with raw data alone, was achieved by the joint modeling of the calibration constant, using our method and the microrheometry data. This was possible for the used microrheometry system presented in (17,18), which can measure viscoelasticity up to the stiffness as found in breast tumor biopsies. Specifically, this system has a high degree of homogeneity in magnetic field gradients within each microscopy FOV, where the system could be modeled with a single calibration constant. Instead, for more nonhomogeneous magnetic field gradients, the proposed calibration procedure would need to be adjusted; for example, it could be extended to allow spatial variations by adding a Gaussian process prior to the volumetric force constant (f_v) in a similar fashion how viscoelastic properties are treated in Eq. 2.

For the method, the inclusion of a measurement error model of the probe radius validated the method for the microrheometry data from the 3D collagen-based experimental samples. Specifically, it showed an expected increase in the uncertainty of the viscoelastic fields as there is more flexibility to fit the probe displacement signals. However, when imaged in unideal optical conditions, for example, in highly thick 3D ECM mimics with no corrections for optical aberrations, the reduced imaging quality may mean that it is unrealistic to obtain an accurate subpixel resolution in the radius estimation consistently. Propagating the error from the radius estimate as carried out in our method is important for avoiding overconfident predictions, especially in measurement conditions where imaging conditions and the imaged dynamics are complex.

We applied the method to quantify the 3D collagen type 1 remodeled by the CAFs of human breast cancer. In general, fibroblasts are known to remodel the collagen via multiple ways (37). With this presented method, we can quantify the spatially varying viscoelasticity in 3D CAF cell culture ECM mimics. We found increased levels of collagen stiffness (as also observed in (12)) that can be accounted to be arising from the CAFs' secretion of ECM components, including fibrillar collagen and fibronectin, which increase the rigidity of the fiber network in the ECM. In turn, the quantified decreased viscous characteristics in this ECM mimic could be due to increased levels of cross-linking in the fiber network, decreasing the energy dissipation by the network under stress (38). Furthermore, the initial high heterogeneity in stiffness (elasticity) in 3D CAF cultures is likely due to the CAFs remodeling of the collagen fibers' network (Fig. S15). These elastic fibers are organized around cells, where there are regions of higher and lower stiffness values. We also found weak evidence of localized changes across FOVs, which could be due to heterogeneous CAF populations (31). These CAFs (from a breast tumor tissue sample) are expected to have cell-to-cell differences, and they may have noticeably different characteristics with respect to abilities to remodel the collagen fiber network.

Concerning magnetic microrheometry, a major limiting factor is a decreased spatial resolution arising from the sparsity of the measurement probes within each microscopy FOV, and the resolution is the main contributor to an increased uncertainty in the spatial viscoelasticity maps. While this method has demonstrated capabilities in quantifying spatial differences and dynamics in ECM mimics with an existing magnetic microrheometer, so far, the method remains incapable of providing information that estimate remodeling of the ECM by individual CAF cells. Previously, such measurements have been done with either using optical tweezers with hundreds of measurement probes and often with the help of collagen fiber staining (12), although that technique cannot obtain data from sufficiently high stiffness levels. From the systems design perspective, this issue could be solved by using smaller magnetic probes that have a higher magnetization, increased spatially varying magnetic fields, and a higher magnification for the camera with maintaining the same FOV. Thus, the proposed method could be used with the same way as described to obtain the spatial viscoelasticity maps. Experimental designs could also be tuned to decrease the number of cells so that the number of probes per cell is simplifying the detection of viscoelasticity differences/gradients. Similarly, correlating the number of cells within a FOV to changes in the viscoelastic properties could be performed by measuring samples with different cell seeding densities (Fig. S16).

This biophysics-based modeling method has the capability of quantifying breast tumor 3D cultures, opening new avenues of modeling research, such as considering more constrained parameterizations for the viscoelastic fields for greater precision. The current choice provides flexibility across multiple measurement conditions but, due to the lack of data points with the sparsity of probes, the information for spatial constraining would likely result in a larger uncertainty. The nonstationary kernel functions or composition of multiple stationary kernels could be a relevant fit for the purpose, but could complicate the MCMC sampling due to the added flexibility. One further interesting direction for continued work could be to consider ideas from fiber networks (39) to provide physically feasible regularization for the spatial viscoelasticity behaviors.

CONCLUSION

We develop a Bayesian modeling method to probabilistically analyze and visualize spatial viscoelasticity in 3D cell culture ECM mimics using magnetic microrheology data for the first time. We use raw displacement signals by the probes in limited numbers in each microscopy FOV, jointly with inclusion of Gaussian processes for the estimation of viscoelastic fields,

providing a flexible means for extracting spatial information of an ECM mimic, appearing heterogeneous in the breast tumor microenvironment. Initially, we calibrate the method together with microrheometry, and obtain a coefficient of variation that dropped to below one tenth of the value obtained using the raw data results only. The results using the calibration are presented in two parts. First, we validated the method's operation for quantifying a controlled stiffness difference. We were able to verify the method's operation by recording a detectable stiffness gradient in the interface of changing concentrations of collagen type 1, which is the most abundant molecule in the tumor tissue. Second, we applied the method to quantify and visualize differences in viscoelasticity within a 3D collagen material that embeds human breast cancer CAF cells, the most abundant cell type in the breast tumor tissue. The CAFs' presence stiffens the collagen material, which aligns with previous research. Importantly, we provided probabilistic quantification of spatial heterogeneity differences in viscoelasticity recorded by magnetic microrheometry, for the first time. The CAFs' culturing leads to an initially higher spatial heterogeneity in collagen stiffness. Overall, we present a method that is capable of enhanced quantification of spatially varying viscoelasticity in breast cancer 3D cultures, with the future potential for enabling matching of spatial viscoelasticity distribution in 3D cultures with the one in biopsies.

DATA AND CODE AVAILABILITY

Research data available at OSF: https://www.doi.org/10.17605/OSF.IO/EAKF2. Software can be found at https://github.com/arasalo1/spatial_microrheology.

ACKNOWLEDGMENTS

J.P. and the other authors acknowledge our project grant from the Jane and Aatos Erkko Foundation during 2024–the present, the Instrufoundation fellow grant from the Instrumentarium Science Foundation during 2021–2024, the distinct Business Finland R2B projects during 2021–2022 and 2024–the present, and the Seed Funding from Aalto University during 2022–2023. We thank Dr Antti Isomäki in the Biomedicum Imaging Unit of University of Helsinki for the imaging of collagen fibers in 3D CAF cultures. We are also thankful to Väinö Mäntylä for help with samples and imaging process.

AUTHOR CONTRIBUTIONS

O.A. and J.P. conceptualized the research. O.A. designed the statistical models. M.H. participated in designing the statistical model specifics, particularly in respect to Gaussian processes. O.A. and J.P. analyzed the results. The experiments were designed by A.J.L., O.A., M.K., and J.P. and carried out by A.J.L. and M.K. The research was led by J.P. A.J.L., O.A., M.K., and J.P. participated in writing the article.

DECLARATION OF INTERESTS

The authors declare no competing interests.

SUPPORTING MATERIAL

Supporting material can be found online at https://doi.org/10.1016/j.bpj.2024.12.010.

REFERENCES

1. Saraswathibhatla, A., D. Indana, and O. Chaudhuri. 2023. Cell–extracellular matrix mechanotransduction in 3D. *Nat. Rev. Mol. Cell Biol.* 24:495–516.
2. Pickup, M. W., J. K. Mouw, and V. M. Weaver. 2014. The extracellular matrix modulates the hallmarks of cancer. *EMBO Rep.* 15:1243–1253.
3. Chaudhuri, O., J. Cooper-White, ..., V. B. Shenoy. 2020. Effects of extracellular matrix viscoelasticity on cellular behaviour. *Nature.* 584:535–546.
4. Acerbi, I., L. Cassereau, ..., V. M. Weaver. 2015. Human breast cancer invasion and aggression correlates with ECM stiffening and immune cell infiltration. *Integr. Biol.* 7:1120–1134.
5. Sun, B. 2021. The mechanics of fibrillar collagen extracellular matrix. *Cell Rep. Phys. Sci.* 2:100515.
6. Chang, J. M., I. A. Park, ..., W. K. Moon. 2013. Stiffness of tumours measured by shear-wave elastography correlated with subtypes of breast cancer. *Eur. Radiol.* 23:2450–2458.
7. Bohte, A. E., J. L. Nelissen, ..., R. Sinkus. 2018. Breast magnetic resonance elastography: a review of clinical work and future perspectives. *NMR Biomed.* 31:e3932.
8. Daviran, M., H. S. Caram, and K. M. Schultz. 2018. Role of cell-mediated enzymatic degradation and cytoskeletal tension on dynamic changes in the rheology of the pericellular region prior to human mesenchymal stem cell motility. *ACS Biomater. Sci. Eng.* 4:468–472.
9. Han, Y. L., P. Ronceray, ..., M. Guo. 2018. Cell contraction induces long-ranged stress stiffening in the extracellular matrix. *Proc. Natl. Acad. Sci. USA.* 115:4075–4080.
10. Jones, C. A. R., M. Cibula, ..., B. Sun. 2015. Micromechanics of cellularized biopolymer networks. *Proc. Natl. Acad. Sci. USA.* 112:E5117–E5122.
11. Keating, M., A. Kurup, ..., E. Botvinick. 2017. Spatial distributions of pericellular stiffness in natural extracellular matrices are dependent on cell-mediated proteolysis and contractility. *Acta Biomater.* 57:304–312.
12. Jagiełło, A., U. Castillo, and E. Botvinick. 2022. Cell mediated remodeling of stiffness matched collagen and fibrin scaffolds. *Sci. Rep.* 12:11736.
13. Furst, E. M., and T. M. Squires. 2017. Microrheology. Oxford University Press.
14. Schultz, K. M., and E. M. Furst. 2012. Microrheology of biomaterial hydrogelators. *Soft Matter.* 8:6198–6205.
15. Waigh, T. A. 2005. Microrheology of complex fluids. *Rep. Prog. Phys.* 68:685–742.
16. Staunton, J. R., W. Vieira, ..., K. Tanner. 2016. Mechanical properties of the tumor stromal microenvironment probed in vitro and ex vivo by in situ-calibrated optical trap-based active microrheology. *Cell. Mol. Bioeng.* 9:398–417.
17. Lehtonen, A. J., O. Arasalo, ..., J. Pokki. 2023. Magnetic microrheometry of tumor-relevant stiffness levels and probabilistic quantification of viscoelasticity differences inside 3D cell culture matrices. *PLoS One.* 18:e0282511.
18. Pokki, J., I. Zisi, ..., O. Chaudhuri. 2021. Magnetic probe-based microrheology reveals local softening and stiffening of 3D collagen matrices by fibroblasts. *Biomed. Microdevices.* 23:1–14.
19. Zhu, M., K. Zhang, ..., Y. Sun. 2020. Magnetic micromanipulation for in vivo measurement of stiffness heterogeneity and anisotropy in the mouse mandibular arch. *Research.* 2020:7914074.
20. Zhu, M., H. Tao, ..., Y. Sun. 2020. Spatial mapping of tissue properties in vivo reveals a 3D stiffness gradient in the mouse limb bud. *Proc. Natl. Acad. Sci. USA.* 117:4781–4791.

21. Rasmussen, C. E. 2004. Gaussian Processes in Machine Learning. Springer Berlin Heidelberg, Berlin, Heidelberg, pp. 63–71.
22. Paul, S., N. Narinder, …, C. Bechinger. 2021. Bayesian inference of the viscoelastic properties of a Jeffrey's fluid using optical tweezers. *Sci. Rep.* 11:2023.
23. Wang, J., T. Li, …, A. T. Zehnder. 2021. Metamodeling of constitutive model using Gaussian process machine learning. *J. Mech. Phys. Solid.* 154:104532.
24. Ziemann, F., J. Rädler, and E. Sackmann. 1994. Local measurements of viscoelastic moduli of entangled actin networks using an oscillating magnetic bead micro-rheometer. *Biophys. J.* 66:2210–2216.
25. Ripley, B. D. 1988. Statistical Inference for Spatial Processes. Cambridge university press.
26. Stan Development Team. 2023. Stan Modeling Language Users Guide and Reference Manual, version 2.28.0. http://mc-stan.org/.
27. Carpenter, B., A. Gelman, …, A. Riddell. 2017. Stan: A probabilistic programming language. *J. Stat. Software.* 76:1.
28. Dillon, J. V., I. Langmore, …, R. A. Saurous. 2017. Tensorflow distributions. Preprint at arXiv. https://doi.org/10.48550/arXiv:1711.10604.
29. Krajina, B. A., B. L. LeSavage, …, S. C. Heilshorn. 2021. Microrheology reveals simultaneous cell-mediated matrix stiffening and fluidization that underlie breast cancer invasion. *Sci. Adv.* 7:eabe1969.
30. Grinnell, F. 2000. Fibroblast–collagen-matrix contraction: growth-factor signalling and mechanical loading. *Trends Cell Biol.* 10:362–365.
31. Sahai, E., I. Astsaturov, …, Z. Werb. 2020. A framework for advancing our understanding of cancer-associated fibroblasts. *Nat. Rev. Cancer.* 20:174–186.
32. Wu, J., Z. Mao, …, C. Gao. 2012. Gradient biomaterials and their influences on cell migration. *Interface Focus.* 2:337–355.
33. Hadden, W. J., J. L. Young, …, Y. S. Choi. 2017. Stem cell migration and mechanotransduction on linear stiffness gradient hydrogels. *Proc. Natl. Acad. Sci. USA.* 114:5647–5652.
34. Srbova, L., O. Arasalo, …, J. Pokki. 2024. Measuring mechanical cues for modeling the stromal matrix in 3D cell cultures. *Soft Matter.* 20:3483–3498.
35. Heilala, M., A. Lehtonen, …, J. Klefström. 2023. Fibrin stiffness regulates phenotypic plasticity of metastatic breast cancer cells. *Adv. Healthcare Mater.* 12:2301137.
36. Sikic, L., E. Schulman, …, J. Pokki. 2022. Nanoscale tracking combined with cell-scale microrheology reveals stepwise increases in force generated by cancer cell protrusions. *Nano Lett.* 22:7742–7750.
37. Najafi, M., B. Farhood, and K. Mortezaee. 2019. Extracellular matrix (ECM) stiffness and degradation as cancer drivers. *J. Cell. Biochem.* 120:2782–2790.
38. Yucht, M. G., M. Sheinman, and C. P. Broedersz. 2013. Dynamical behavior of disordered spring networks. *Soft Matter.* 9:7000–7006.
39. Broedersz, C. P., and F. C. MacKintosh. 2014. Modeling semiflexible polymer networks. *Rev. Mod. Phys.* 86:995–1036.

Biophysical Journal, Volume *124*

Supplemental information

Probabilistic analysis of spatial viscoelastic cues in 3D cell culture using magnetic microrheometry

Ossi Arasalo, Arttu J. Lehtonen, Mari Kielosto, Markus Heinonen, and Juho Pokki

S1 Supplemental methods

S1.1 Cell viability

We quantified the viability of the initial measurement condition after 1 day of incubation, using Cyto3D Live–Dead Assay. The cells' viability is $96.6 \pm 2.2\%$ (mean $\pm$ standard deviation, 5 measurements with altogether 3603 cells, and 721 cells/measurement on average). We have subsequently controlled that, in the following conditions (after 2 and 3 days), the cells remain spread with almost no exceptions (indicating continued fibroblast viability).

S1.2 Magnetic microrheometry-related biophysics

The magnetic-probe displacement is related to linear viscoelasticity as in the following equation described in [1].

$$6\pi r\eta\dot{x}(t) + 6\pi r\mu x(t) = M\nabla BV = V\underbrace{M\nabla B_0}_{f_v}\sin(\omega t) \tag{S1}$$

$$x(t) = \frac{2f_v r^2}{9|G^*|}sin(2\pi ft - \phi) \tag{S2}$$

where x is the probe displacement, $\dot{x}$ is the probe velocity, r is the probe radius, η is dynamic viscosity, μ is the shear modulus, M is the probe magnetization, V is the probe volume and ∇B is the magnetic field gradient strength. We have applied time-dependent sinusoidal forces with an amplitude of ∇B_0. For convenience, the probe magnetization and the unknown amplitude are fused into a single calibration constant, the volumetric force, f_v, which is estimated during the system calibration. Solving the differential equation in Eq. S1 results in Eq. S2. Instead of estimating the values of η and μ, we have estimated the complex shear modulus, $G^* = G' + iG''$, where the storage modulus is $G' = \mu$, and the loss modulus is $G'' = \omega\eta$. In this work, we have reported the absolute complex shear modulus $|G^*|$ as a measure of stiffness, and the phase angle ϕ of the complex shear modulus, as a measure of viscous energy dissipation (i.e. the liquid-like characteristics).

S1.3 Bayesian modeling method for estimation of spatially varying viscoelasticity

Here, the hierarchical prior for the noise term (σ_i), which is independent for each magnetic probe, is defined:

$$\sigma_\mu \sim \mathcal{N}(0, 1) \tag{S3}$$

$$\sigma_\sigma, l_\sigma \sim \text{Half-}\mathcal{N}(0, 1) \tag{S4}$$

$$\sigma_i \sim \text{Inverse Gamma}(\sigma_\mu, \sigma_\sigma) \tag{S5}$$

Then, the hierarchical prior for the mean behavior, where we use non-centered parametrization for the viscoelasticity terms, is defined for the absolute complex shear modulus and the phase angle:

$$\mu_{|G*|} \sim \mathcal{N}\left(\mathcal{N}(50, 15), \text{Half-}\mathcal{N}(0, 1)\right) \tag{S6}$$

$$\mu_\phi \sim \mathcal{N}\left(\mathcal{N}(0, 0.1), \text{Half-}\mathcal{N}(0, 1)\right) \tag{S7}$$

Table S1: Number of measurements in different conditions for 3D CAF-cell cultures

Condition	# Repetition	Incubation [# Day]	Probes	Fields of view
CAF	1	1	39	7
Contr	1	1	34	4
CAF	1	2	56	7
Contr	1	2	34	4
CAF	1	3	44	6
Contr	1	3	20	3
CAF	2	1	45	6
Contr	2	1	51	6
CAF	2	2	46	6
Contr	2	2	58	6
CAF	2	3	60	6
Contr	2	3	55	6
CAF	3	1	51	6
Contr	3	1	54	6
CAF	3	2	38	6
Contr	3	2	63	6
CAF	3	3	39	6
Contr	3	3	60	6

S1.4 System calibration

The full calibration model is used as follows:

$$x_i(t) \sim \mathcal{N}\left(-\frac{2}{9\eta\omega}r^{*2}f_v\left(\cos(\omega t - \phi_i) - 1\right) + \alpha_i t + \beta_i, \sigma\right) \tag{S8}$$
$$\sigma \sim \text{Inverse Gamma}(\alpha = 5, \beta = 5) \tag{S9}$$
$$r_\mu \sim \mathcal{N}(6, 1) \tag{S10}$$
$$r_\sigma \sim \text{Inverse Gamma}(\alpha = 2, \beta = 0.5) \tag{S11}$$
$$r^* \sim \mathcal{N}(r_\mu, r_\sigma) \tag{S12}$$
$$r \sim \mathcal{N}(r^*, \tau) \tag{S13}$$
$$f_v \sim \mathcal{N}(280000, 14000) \tag{S14}$$
$$\alpha, \phi \sim \mathcal{N}(0, 0.1) \tag{S15}$$
$$\beta \sim \mathcal{N}(0, 3.) \tag{S16}$$
$$\eta = 1.000074433378914 \tag{S17}$$
$$\omega = 0.05(2\pi) \tag{S18}$$

The calibration model has the same formula as described in the main article text with the addition of the parameter ϕ to handle small miscalibrations, and the letters α_i and β_i to capture small linear drifts in the data. The integration constant C is found using the initial guess of $x(t = 0) = 0$.

The prior for f_v, the primary output of the calibration model, shows minimal sensitivity to prior choices. As long as the mean and scale of the prior cover the true value, all choices converge to the same result, well within the standard deviation reported in the main text.

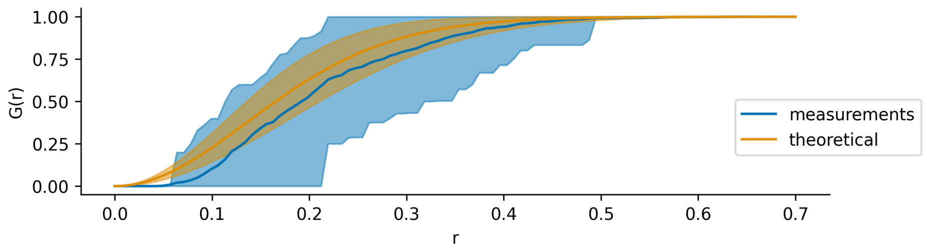

Figure S1: Ripley's G-function calculated for the magnetic probe locations. Comparison is done against homogeneous Poisson point process $G(r) = 1 - e^{-\lambda\pi r^2}$ (where $\lambda = n/A$, n is number of cells in a FOV and A is the area of a FOV and r is the normalized distance). Intervals clearly overlap meaning no strong indications of clustering or aggregation.

Further details on prior sensitivity of τ are included in the subsection S2.1. These results also apply to the current calibration model.

S2 Supplemental results

S2.1 Sensitivity analysis

We performed a sensitivity analysis to test how different priors affect the model's results. Our focus was on the priors most important for stability of the viscoelastic field estimates and the radius correction. By varying these priors, we evaluated their impact on the stability of the results.

Correctly inferring posteriors for the magnitude of heterogeneity (α_μ) and heterogeneity differences between FOVs (α_σ) in $|G^*|$ fields is important from the perspective of interpretability. Too restrictive prior choices (wrongly informative) can lead to incorrect interpretation of the parameters while still providing stable estimates for the viscoelastic fields shown in the left column of Fig. S2. The top row in the figure (Fig. S2A) shows the FOV-specific magnitude of the heterogeneity with the global mean and standard deviation removed ($\alpha_{z[i]}$ in non-centered parametrization $\alpha_{[i]} = \text{Softplus}(\alpha_\mu + \alpha_\sigma \alpha_{z[i]})$). This $\alpha_{z[i]}$ shows how too low α_μ values result in a positive bias. Now, too low global magnitude, in Fig. S2B, is partially explained by the bias and increased FOV variability (α_σ). When the scale parameter of the priors are increased the true region of the heterogeneity is covered properly and differences in the prior choices become unsensitive. We also verified if changes in the hyperprior of the $\mu_{|G^*|}$ scale affects the inference. In theory, this parameter can lead to oversmoothing in the Gaussian process, because the spatial variability could be explained by highly varying mean level having a large uncertainty. However, as evident from the rightmost column in Fig. S2, that change is redundant as it does not affect α_μ or α_σ noticeably.

Hence, as long as the chosen prior has thick enough 'tails' to capture the true scale of the heterogeneity, in our case in the order of 10 Pascals, model becomes unsensitive to the prior choices. Student's $\text{t}(3, 0, 20)$ for the α_μ with $\mathcal{N}(0, 5)$ for α_σ have scale parameters which are applicable to capture the heterogeneity of the measured collagen matrices.

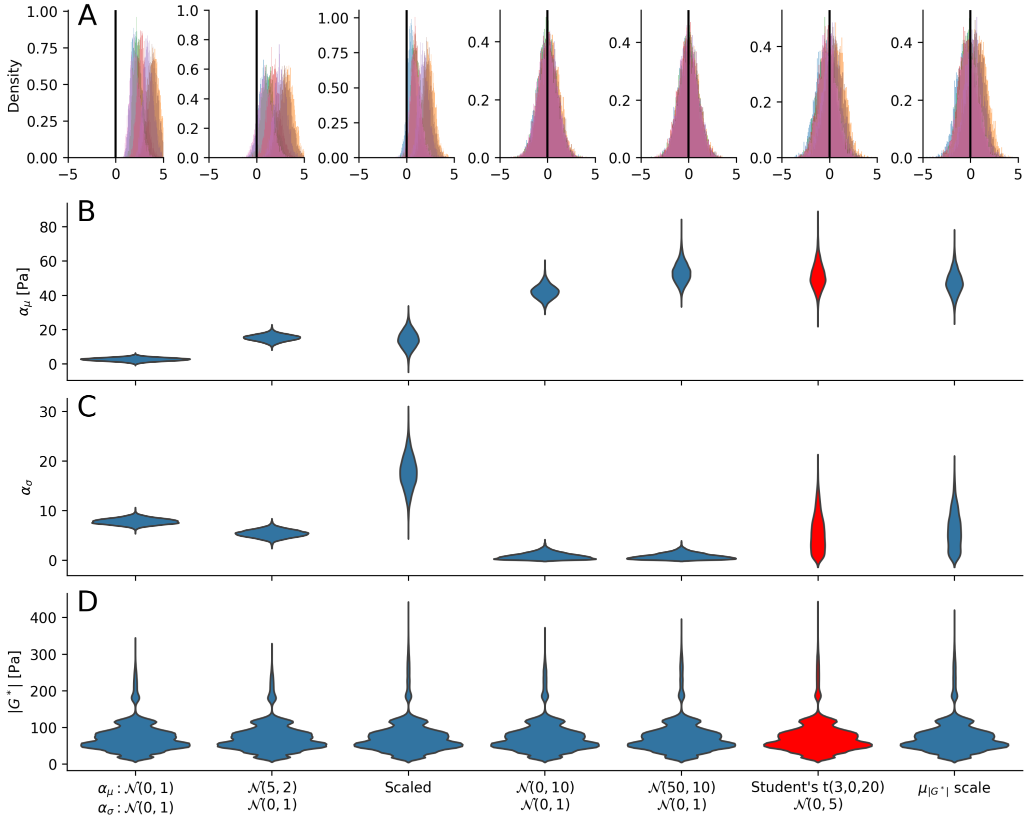

Figure S2: Evaluation of the tested priors is visualized, and the chosen priors are indicated with red color. **(A)** Posterior distributions of $\alpha_{\mathrm{z[j]}}$ with different priors for α_μ and α_σ. A positive bias is induced if the prior is too restrictive that is visible clearly for $\mathcal{N}(0,1)$. Instead, the priors with longer 'tails' for α_μ (e.g. $\mathcal{N}(0,10)$ or $\mathcal{N}(50,10)$) lead to the desired $\alpha_{z[j]}$ remaining at zero and having no bias. In 'Scaled', a multiplication by 10 is applied to the original priors ($\alpha_\mu \sim \mathcal{N}(0,1)$ and $\alpha_\sigma \sim \mathcal{N}(0,1)$). In '$\mu_{|G^*|}$ scale', hierarchical hyperprior for the scale of mean level, $\mathcal{N}(0,1)$ is increased to $N(0,5)$. **(B)** Identifiably of α_μ (magnitude of heterogeneity) with the different priors. When the scale increases the value is identified correctly. **(C)** Effects of the priors to the heterogeneity between microscopy fields of view (FOVs). **(D)** Posterior of $|G^*|$ across over all locations and spatial points. While the interpretation changes, estimated viscoelastic field stays similar suggesting the model is robust to different choices of priors.

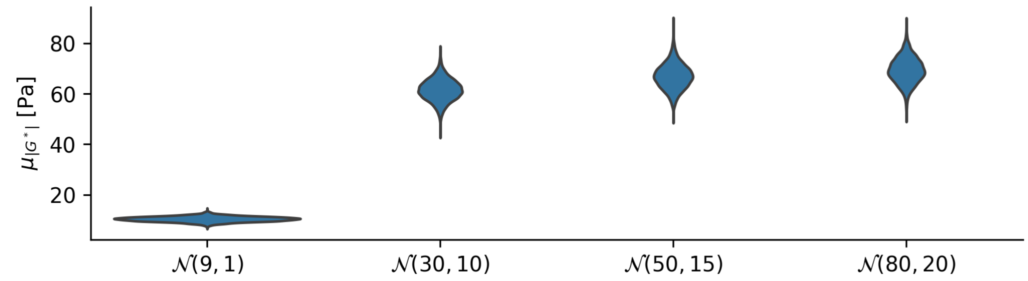

Figure S3: Effects of the prior choice in $\mu_{|G^*|}$. The model is robust unless the prior is highly misspecified as in $\mathcal{N}(9, 1)$.

Kernel length scale for the $|G^*|$ field does not suffer from similar challenges in the interpretability as we are more interested in good fit rather than understanding the dynamics in the light of length scale. In short, length scale is more limited by the distance of the magnetic probes. If we have multiple FOVs with different 'closeness' of the probes, the interpretability is difficult as the FOVs with more spread out probe distribution has less information about the fast variations in the material. Therefore, comparison becomes difficult. The choice of the hierarchical prior is also not important as long as it captures the size of the measurement window. The coordinates are normalized between $[0, 1]$, so the choice of Half-$\mathcal{N}(0, 1)$ captures possible variation for both $|G^*|$ and ϕ.

We also tested how the model behaves if we change the prior for the $|G^*|$ offset $\mu_{|G|^*}$. The results are visible in Fig. S3, which shows that the choice of $\mathcal{N}(50, 15)$ is reasonable as the actual data mean is within the prior. If this is highly misspesified, such as $\mathcal{N}(9, 1)$, estimated mean level is simply wrong and the interpretation of the value would be different. In that case to be avoided, the mean of the data is partially explained by the amplitude of the Gaussian processes which is unwanted behavior for interpretability. Obviously, if this model would be used for stiffer materials, the mean prior should be scaled accordingly to contain the true mean or alternatively the whole amplitude could be scaled based on the data so more 'default' priors could be used.

The phase angle ϕ, the second viscoelasticity parameter, is insensitive to the choices of priors for the kernel amplitude parameter. The main reason for this is that the ϕ's domain is finite ($[0, \pi/2]$), which is not the case for the domain of $|G^*|$ (i.e. $\mathbb{R}^+$). Thus, the possible heterogeneity of ϕ is limited to

a smaller range which can be covered with simple choices such as $\mathcal{N}(0, 0.5)$. We found that if the prior is far away from zero (positive or negative), the Sigmoid function squashes the values and too much mass is put to either having a zero heterogeneity, or toward the absolute maximum. MCMC can slow down considerably and computational diagnostics can report divergent transitions indicating poor exploration of the parameter space. A possible reason for such behaviors is the promotion of unrealistically large values which are close to the saturating parts of the Sigmoid function. This behavior is visible if the scale is ≥ 1.

On the other hand, for the mean level μ_ϕ, we iteratively test different choices of priors shown in Fig. S4. Posterior distributions of ϕ fields remain unaffected the but interpretability of specific parameters can be changed if the prior is poorly chosen. Narrow priors such as $\mathcal{N}(0, 0.1)$ are too restrictive and might not capture the true mean value and result in increased scale of the mean level (Fig. S4B). Additionally, the transformation $f(x) = \arcsin(\text{Sigmoid}(x))$ used to map the real axis to $[0, \pi/2]$ transform the mean of 0 to ≈ 28 degrees which means the prior is biasing towards unintended value. Increasing the scale captures the true value as visible in Fig. S4A.

As pointed out in the main text, we want to promote elastic like properties because collagen cultures are mostly elastic. This can be achieved by giving negative mean values, because they are transformed to approximately 0. As shown in S4, these choices are irrelevant in identifying the parameters. The prior recommended in the main text is chosen because of the ease in interpretation. Here the transformation of the parameter is characterized by the prior choice of $\mathcal{N}(0, 0.3)$, meaning we are defining the prior in the transformed space. This choice is purely for the benefit of understandability, it does not have any practical implications as shown in Fig. S4. Similarly, if we would like to promote viscous properties the prior could be changed to $\mathcal{N}(\pi/2, 0.3)$ which is difficult to define in the original space.

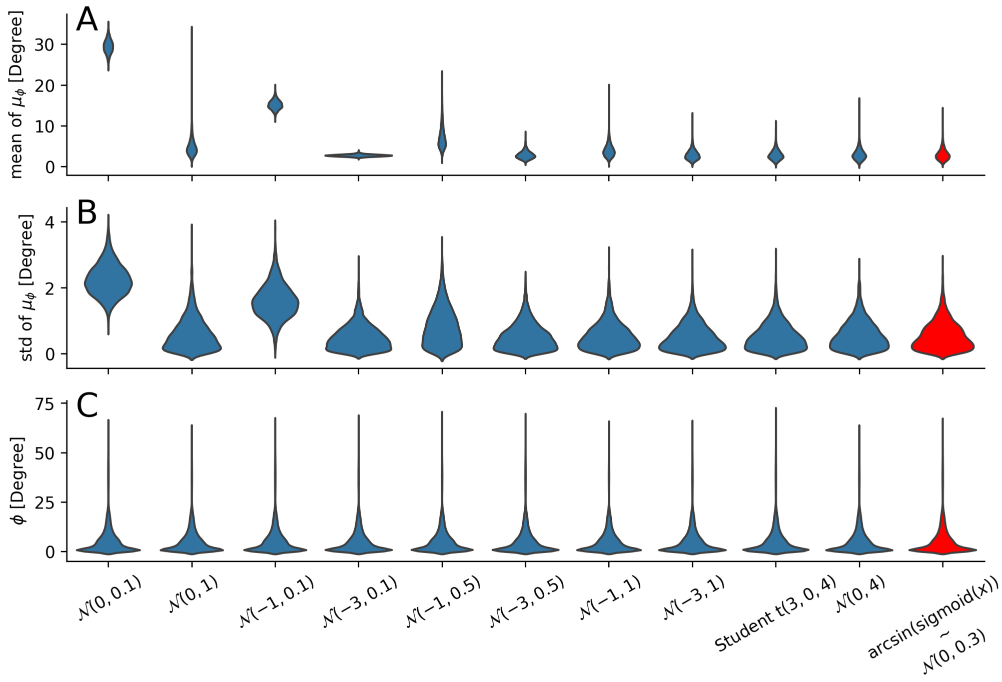

Figure S4: Sensitivity of the estimated phase angle's (ϕ) mean level to priors of the mean level. The estimated standard deviation of the mean level and ϕ fields are also shown. (**A**) Posterior of the mean-level parameter μ_ϕ. As long as the width of the distribution is sufficient, the correct level is inferred insensitively. (**B**) shows how misspecifications lead to positive bias in the mean standard deviation parameter. (**C**) Posterior distributions remain unaffected as a function of the priors. The red color highlights the updated choice of the prior (arcsin(sigmoid(μ_ϕ)) $\sim \mathcal{N}(0, 0.3)$), based on the sensitivity analysis. All priors except for the updated prior are defined in the untransformed space. Some of the priors have therefore negative mean values as they are transformed to the domain of ϕ after sampling.

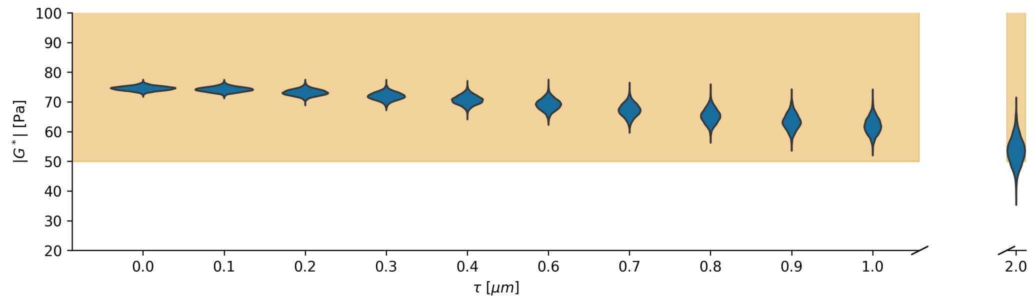

Figure S5: Sensitivity of modeled $|G^{*}|$ to varied τ. Blue violinplots indicate posterior distributions of the mean. The orange shading shows the region between the data's 25th to 75th percentiles.

Next, we focus on the sensitivity analysis of r^{*} to τ as it controls how large errors are allowed which in turn can overly smooth the results due to squared relation to the amplitude of the signal. We fit the model at values between $[0, 2]$ which equal at maximum of $\approx \pm 6$ pixels of error to a one representative measurement condition to evaluate variability. As evident from the Fig. S5A, increasing τ increases the uncertainty (width of the distributions) while decreasing the mean. At large values of τ the model has more flexibility to solve the inverse problem and due to the squared relationship of the radius estimate, spatial variability in the viscoelasticity is now explained by highly varied magnetic bead size. Finally, as τ increases to unrealistically large values, the model approaches $|G^{*}|$ prior $\mu_{|G|*} \sim \mathcal{N}(50, 15)$.

The choice of 0.1 used in this paper is reasonable as it is insensitive to changing the radius values, while still capturing possible uncertainty in the radius estimate. However, as shown, this is an influential parameter so care must be taken. For example, domain expertize of the used imaging system shouldn't be downplayed either. If the imaging quality is poor (large τ), or alternatively, probe size would be much smaller (larger contribution from τ), this parameter would dominate the results and the final spatial fields would be smoother. In a way this is also useful behavior as it reflects the uncertainty of the measurement system more accurately, and should raise concerns of applicability of the measurement device and the collected data. In our case the choice of 0.1 reflects our prior understanding of the radius estimate accuracy in our imaging system and is shown to have only a small effect to the final results.

S2.2 Posterior checks

Figure S9 shows how the model differs from the traditional data analysis pipeline. In Fig. S9A,B we see how the model and the data (traditional analysis pipeline) follow each other closely. However, the uniqueness of different FOVs (violinplots' irregular shapes) is more pronounced as the model is less subject to noise in measurements and captures viscoelasticity more robustly.

Figure S9C indicates how much the model is recalibrating the uncertain radius values. As specified by the prior choice of 0.1 for τ, changes are small. Further, the shape is symmetric suggesting that the radius estimates are not biased which gives some further evidence that the algorithm for calculating the radius values is working similarly across samples.

The level of difference between the model and the traditional analysis pipeline are shown in Figs. S9D and E. Differences are mostly in the range of few Pascals and $\pm$ 2 degrees. These findings further support the findings in Figs. S9A and B how to model matches closely what we are expecting to get from the data.

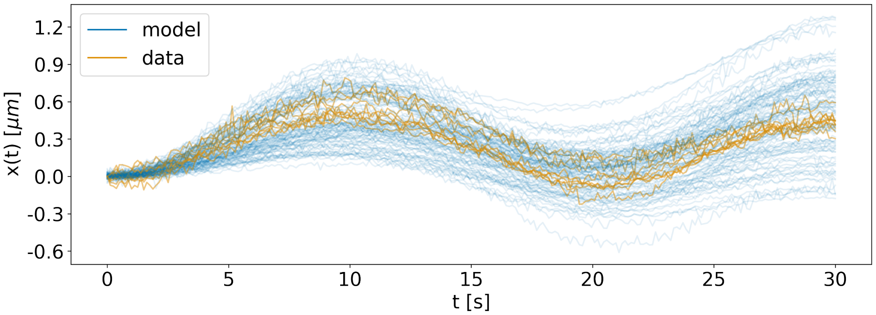

Figure S6: Draws from the calibration model's prior distribution, compared to the raw probe displacement data.

A more detailed visualization of the differences between the model and the traditional analysis pipeline are shown in Fig. S10. It shows how the inferred viscoelastic properties are similar to the averages of different magnetic-reference probe pairs, which is the intended behavior. Further, large outliers are mostly rejected resulting in longer tails (higher uncertainty). If the number of reference probes is only one, we see more averaging behavior with wider distributions. As the model has less information for that particular probe it relies more on the pooled information from the hierarchical priors and shape of the field generated by the Gaussian process.

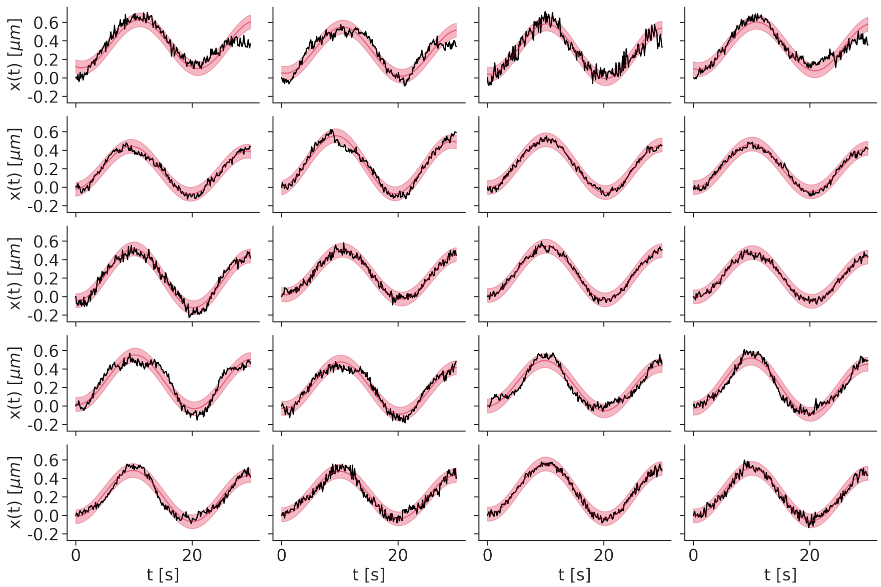

Figure S7: Posterior predictive checks of the measured probe displacement signals in the calibration data (showing half of the signals). Shaded regions represent the 95% credible intervals.

S2.3 Additional results

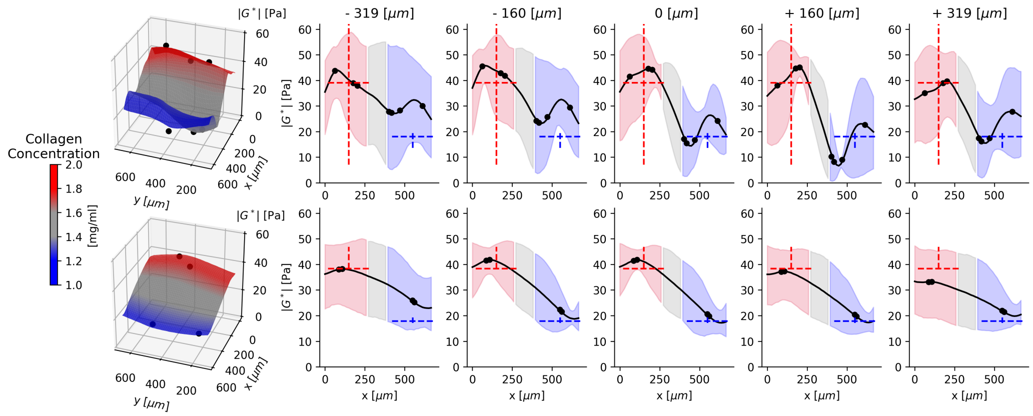

Figure S8: Supplementary visualization of stiffness gradient at varied off-axis positions, relative to the centerpoint of the y axis, in Fig. 3 of the manuscript.

Table S2: Probabilistic differences in viscoelasticity's mean levels ($\mu_{|G^*|}$, and μ_ϕ) in comparisons between each 3D cultures condition and its controls. Shaded rows show the differences over all repetitions in a condition. Values as bolded indicate significance using a threshold value of 0.95.

Incubation day	Repetition ID	Stiffness [%] $P(\mu_{\|G^*\|}^{\text{CAF}} > \mu_{\|G^*\|}^{\text{control}})$	Phase angle [%] $P(\mu_\phi^{\text{CAF}} < \mu_\phi^{\text{control}})$
1	1	**99.75**	58.42
1	2	**99.98**	**96.4**
1	3	**98.15**	**97.4**
1		**99.29**	84.08
2	1	**99.08**	73.07
2	2	**99.78**	47.97
2	3	**99.95**	**99.92**
2		**99.60**	73.66
3	1	13.53	82.82
3	2	85.83	54.02
3	3	**99.93**	93.35
3		66.43	76.73

Table S3: Probabilistic differences in viscoelasticity's mean levels ($\mu_{|G^*|}$, and μ_ϕ) in comparisons between different days of incubation for the 3D cultures.

Incubation days	Stiffness [%] $P(\mu_{\|G^*\|}^{\text{CAF day n}} < \mu_{\|G^*\|}^{\text{CAF day n+1}})$	Phase angle [%] $P(\mu_\phi^{\text{CAF day n}} < \mu_\phi^{\text{CAF day n+1}})$
1 & 2	71.94	66.08
1 & 3	74.73	67.86
2 & 3	52.76	47.32

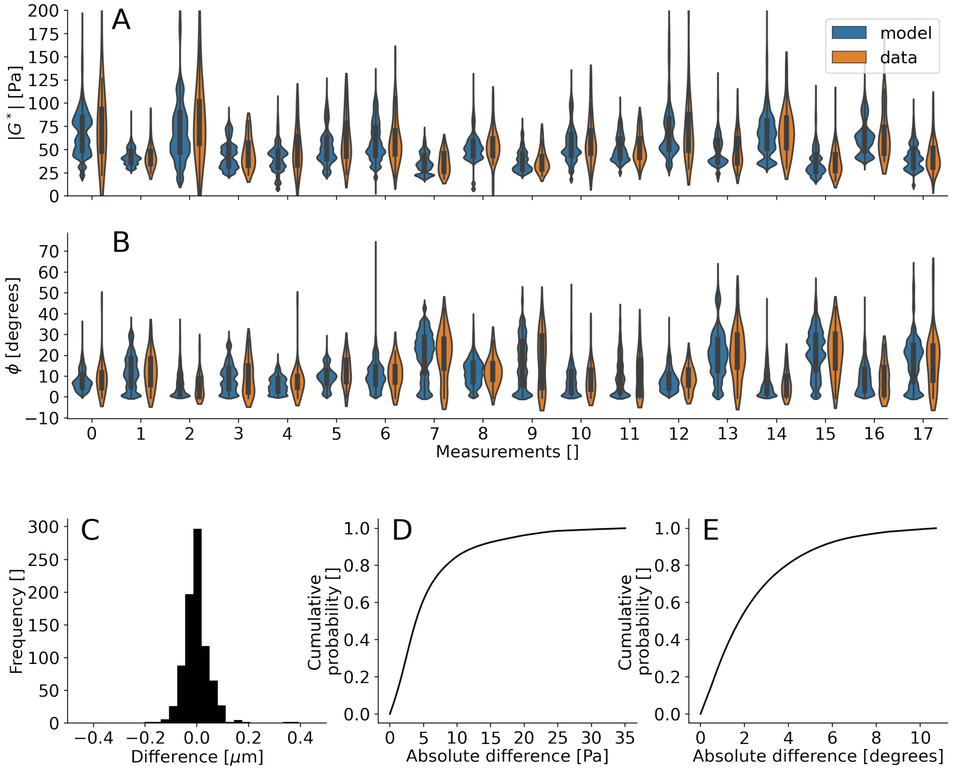

Figure S9: Posterior validation. **(A)** and **(B)** show a comparison of estimated viscoelastic properties calculated from the raw measurements and the model. The different sets of measurements, noted as 'Measurements' in the X axes (with all magnetic probes over multiple FOVs within a single violinplot) are shown. **(C)** shows the magnitude of the radius adjustments done by the measurement error model. **(D)** and **(E)** show the cumulative empirical probability distributions of the absolute differences in the viscoelastic estimates (raw data and the model) for the absolute complex shear modulus and the phase angle, respectively.

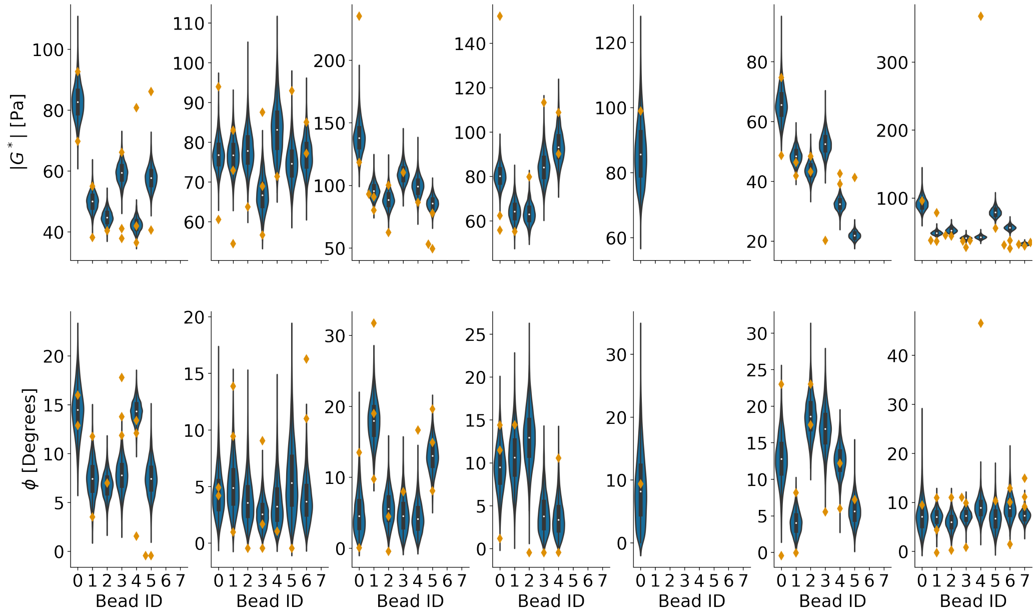

Figure S10: Comparison of estimated viscoelastic properties calculated from the raw measurements and the model outputs from a single sample holder for measurements. Orange triangles represent estimates calculated using conventional pipeline, and blue violin plots show the same value estimated with the Gaussian processes. Different columns are different measured FOVs and the X axes distinguish the different used magnetic probes.

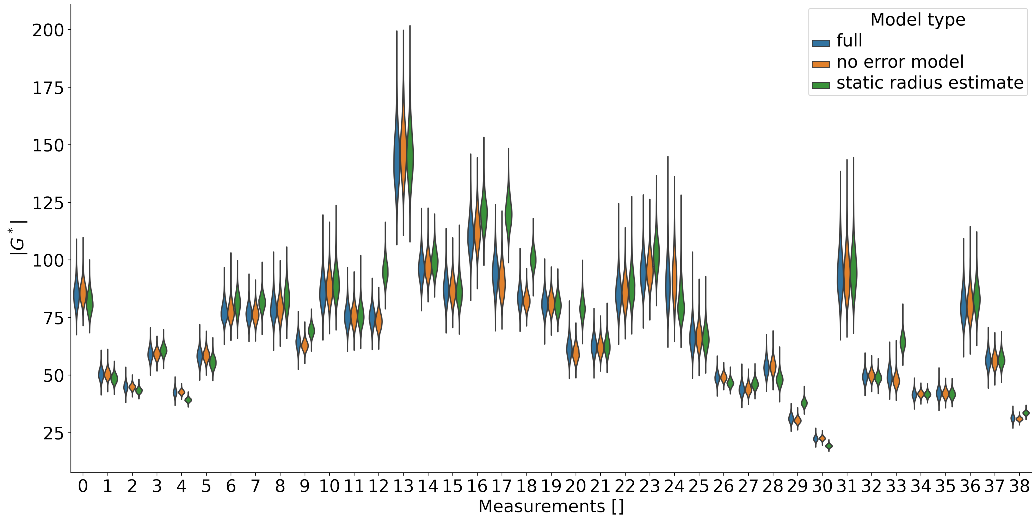

Figure S11: Model comparisons in respect to inclusion of the radius estimation in the model. The X axis shows different measured probes and the Y axis indicates the absolute complex shear modulus. Different colors show different versions of the spatial model. The 'full' model is the proposed model, the 'no error model' uses the raw measurements instead of the measurement error model, and the 'static radius estimate' uses the mean probe radius of 6.14 μm. The changes in the model are highly overlapping indicating that the measurement error model does not shift values unrealistically.

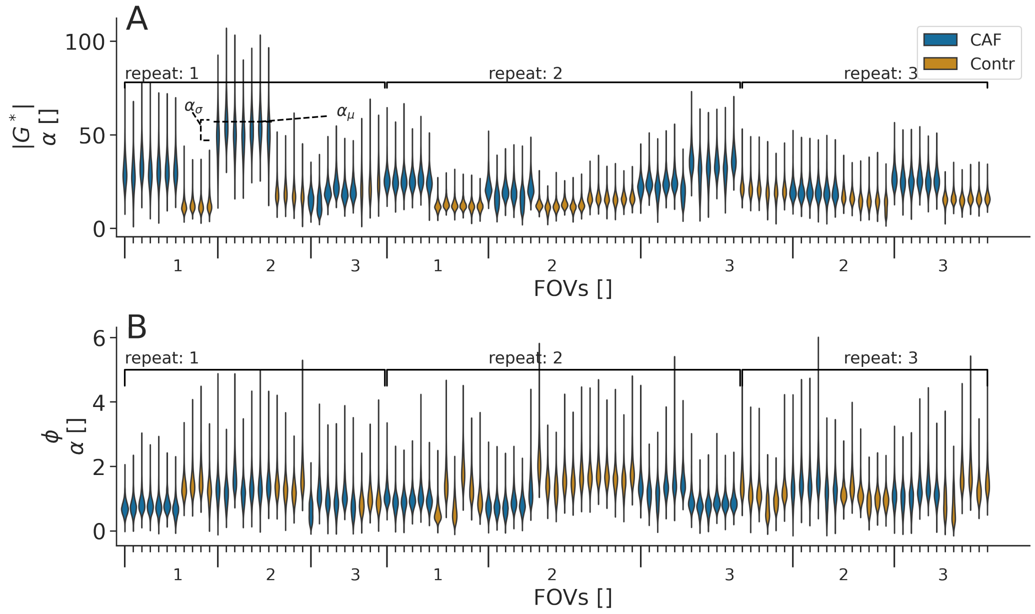

Figure S12: Estimate of heterogeneity in viscoelasticity in respect to varied microscopy FOV. Y axes show the magnitude of the heterogeneity in the **(A)** absolute complex shear modulus and the **(B)** phase angle. Minor ticks in the X axis represent the individual FOVs while the major ticks are corresponding the incubation day number. The meanings of α_μ and α_σ for single measurement conditions are depicted visually.

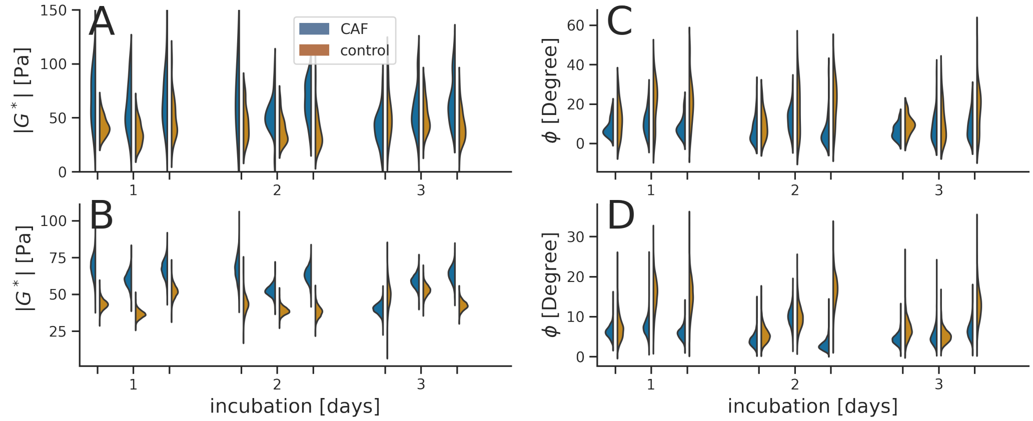

Figure S13: Posterior distributions for each measurement condition plotted. A and C are the full posteriors while B and D are the mean offsets. Minor ticks indicate different repeations.

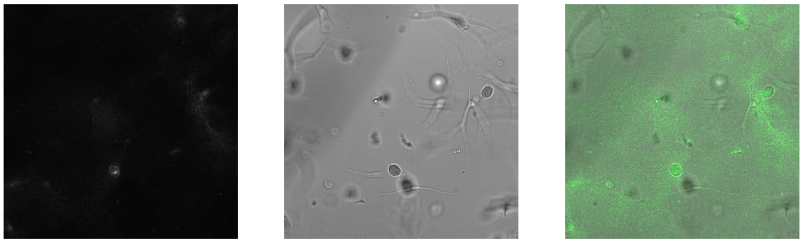

Figure S14: Unprocessed images of collagen fibers (reflectance, left) and breast-cancer-associated fibroblasts (brightfield, middle), and these images having overlaid (right). The overlaid image has been postprocessed using contrast limited adaptive histogram equalization (CLAHE). The images' width/height is 581 μm.

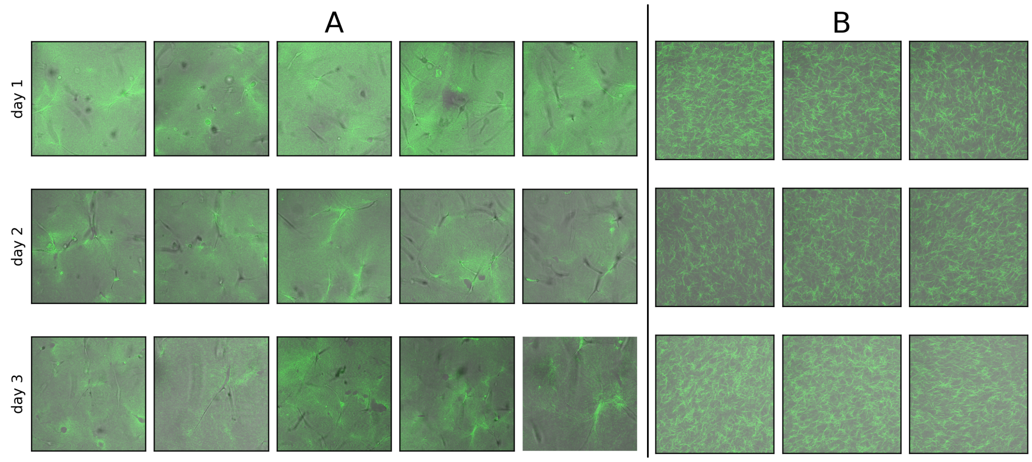

Figure S15: Imaged collagen-fiber network in respect to the cancer-associated fibroblasts. Microscopy fields of view (FOVs) on collagen fibers (reflectance) and fibroblast locations (brightfield) are overlaid. **(A)** Overlaid typical images on collagen-based 3D fibroblast cultures over incubation of 1, 2, and 3 days. For each incubation time, there are duplicate 3D culture samples. **(B)** Control collagen samples for the varied incubation times. There are differences between signal strengths depending on the imaging time for each incubation condition (day 1–3), indicating that the presence of collagen fibers rather than the strength of fibers' reflectance signal is relevant in this data. NB: All the images have been postprocessed using CLAHE. The images' width/height is 581 μm.

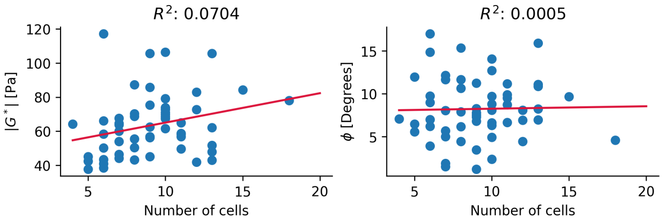

Figure S16: Estimated viscoelasticity (stiffness $|G^*|$ and phase angle ϕ) as a function of cell number. Specifically, the relation between $|G^*|$ and the cell number has a significant p-value <0.05 for the slope, but the coefficient of determination is only roughly 0.07 meaning that the number of cells predicts poorly the variation of the data. Therefore, this correlation is highly uncertain.
